# Development of Pyrazolopyrimidine Anti-*Wolbachia* Agents for the Treatment of Filariasis

**DOI:** 10.1021/acsmedchemlett.1c00216

**Published:** 2021-08-20

**Authors:** Paul McGillan, Neil G. Berry, Gemma L. Nixon, Suet C. Leung, Peter J. H. Webborn, Mark C. Wenlock, Stefan Kavanagh, Andrew Cassidy, Rachel H. Clare, Darren A. Cook, Kelly L. Johnston, Louise Ford, Stephen A. Ward, Mark J. Taylor, W. David Hong, Paul M. O’Neill

**Affiliations:** † Department of Chemistry, University of Liverpool, Crown Street, Liverpool L69 7ZD, United Kingdom; ‡ Department of Tropical Disease Biology, Liverpool School of Tropical Medicine, Pembroke Place, Liverpool, L3 5QA, United Kingdom; § Drug Safety & Metabolism, IMED Biotech Unit, AstraZeneca U.K., Cambridge CB2 0AA, United Kingdom; ∥ Oncology Safety Sciences, Clinical Pharmacology & Safety Sciences, R&D, AstraZeneca, Cambridge CB2 0AA, United Kingdom; ⊥ Institute of Systems, Molecular & Integrative Biology, School of Life Sciences, University of Liverpool, Crown Street, Liverpool L69 7ZB, United Kingdom

**Keywords:** *Wolbachia*, Pyrazolopyrimidine, Onchocerciasis, Filariasis

## Abstract

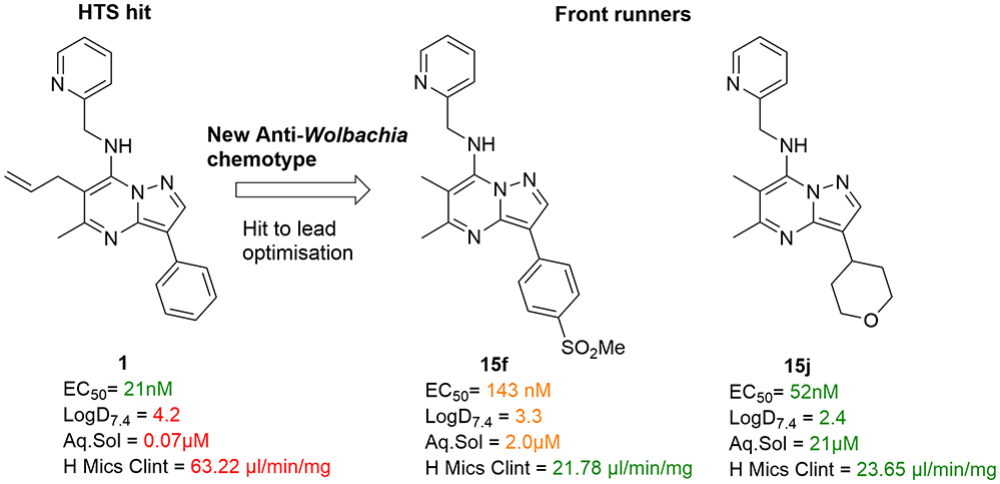

Anti-*Wolbachia* therapy has been identified as a viable treatment for combating filarial diseases. Phenotypic screening revealed a series of pyrazolopyrimidine hits with potent anti-*Wolbachia* activity. This paper focuses on the exploration of the SAR for this chemotype, with improvement of metabolic stability and solubility profiles using medicinal chemistry approaches. Organic synthesis has enabled functionalization of the pyrazolopyrimidine core at multiple positions, generating a library of compounds of which many analogues possess nanomolar activity against *Wolbachia in vitro* with improved DMPK parameters. A lead compound, **15f**, was selected for *in vivo* pharmacokinetics (PK) profiling in mice. The combination of potent anti-*Wolbachia* activity in two *in vitro* assessments plus the exceptional oral PK profiles in mice puts this lead compound in a strong position for *in vivo* proof-of-concept pharmacodynamics studies and demonstrates the strong potential for further optimization and development of this series for treatment of filariasis in the future.

Filarial nematodes are the causative pathogens of the neglected tropical diseases lymphatic filariasis (LF) and onchocerciasis that affect tens of millions people throughout the tropics and contribute to serious public health and socio-economic problems. Onchocerciasis (the cause of river blindness) is the second leading infectious cause of blindness. These diseases combined are one of the leading causes of morbidity worldwide. The main causative agents for these conditions are the nematodes *Onchocerca volvulus* (onchocerciasis), *Wuchereria bancrofti*, *Brugia malayi*, and *Brugia timori* (LF).^1^ The latest recommended treatment for LF in areas which are not coendemic for onchocerciasis or other filarial disease, loiasis, is a triple combination of ivermectin, diethylcarbamazine plus albendazole.^2^ Ivermectin is the recommended treatment for onchocerciasis; however, it cannot be used in areas co-endemic for loiasis due to potentially fatal adverse effects. These direct acting antifilarial drugs primarily target microfilariae, the immature worm stage, and thus can prevent transmission, but they have little macrofilaricidal activity against the adult worms. Hence, these drugs require lengthy treatments that can be as long as 15 years.^3,4^ The association of current direct acting antifilarial agents with undesired adverse effects, contraindicated patient groups combined with a growing concern of resistance development, is driving current research efforts to identify and generate safe therapeutic alternatives.^5,6^

The nematodes which are responsible for causing these two filarial diseases share an essential endosymbiotic relationship with the bacterium *Wolbachia.*(7,8) Although the exact nature of this relationship is not yet fully understood, anti-*Wolbachia* therapy has been proven clinically by an existing antibiotic, doxycycline, which delivers safe macrofilaricidal activity with superior therapeutic outcomes compared to current antifilarial drugs.^9−11^

Pyrazolopyrimidine compounds have frequently appeared in the literature with a variety of different pharmacological activities such as kinase inhibitors,^12,13^ antituberculosis,^14^ antimalarial^15^ and antiviral^16^ agents, and antidepressants.^17^ The original hit for this chemotype (**1**) which resulted from a phenotypic high-throughput screen (HTS) of a divergent chemical library donated by the Medicines for Malaria Venture (MMV) is displayed in Figure 1 with measured *in vitro* activity (EC_50_) against *Wolbachia* (*w*AlbB) infected insect cells (*Aedes albopictus*, C6/36) and drug metabolism/pharmacokinetic (DMPK) properties. According to the HTS data of other close pyrazolopyrimidine analogues screened in the same campaign (data not shown), some preliminary indication of structure–activity relationship (SAR) was observed and the areas of focus for SAR studies and optimization are highlighted in Figure 1. The aim of the work described here was to develop pyrazolopyrimidine leads with potent anti-*Wolbachia* activity and desired DMPK properties that could be further developed as oral drugs for the treatment of filariasis.

**Figure 1:**
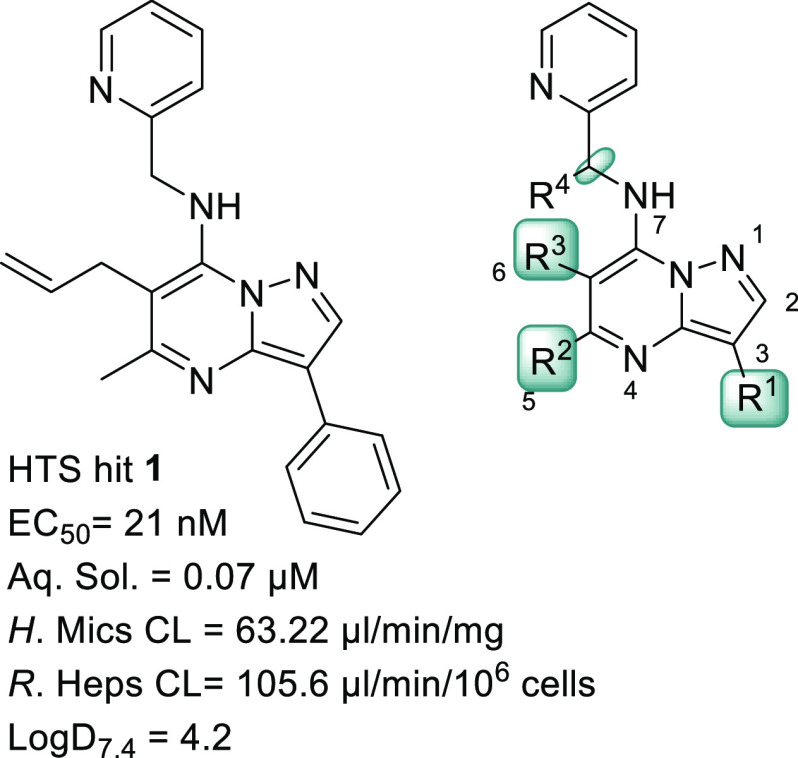
Structure of the HTS hit **1** and the pyrazolopyrimidine scaffold with the areas for SAR studies highlighted.

The cascade depicted in Figure 2 was followed for the optimization of the compounds described in this work. *In vitro* anti-*Wolbachia* potency was assessed in parallel with *in vitro* DMPK screening.^18^ Compounds showing a good balance of potency and metabolic stability were then selected to test their anti-*Wolbachia* activity against *Brugia malayi* microfilariae (mf) *in vitro* and for *in vivo* PK profiling in mice. This secondary *in vitro* assay was developed to provide a link between the *in vitro* insect cell-based assay and the *in vivo B. malayi* SCID mouse model. Although the throughput of the mf assay is limited due to the availability of the *B. malayi* mf, it enables the assessment of the anti-*Wolbachia* activity in one of the targeted human parasites, providing important evidence of translation between different species of *Wolbachia* and hosts. Finally, front runners identified from the two above tests would be selected for the *in vivo* PD study where SCID mice are infected with the human parasite *B. malayi*.^19^

**Figure 2:**
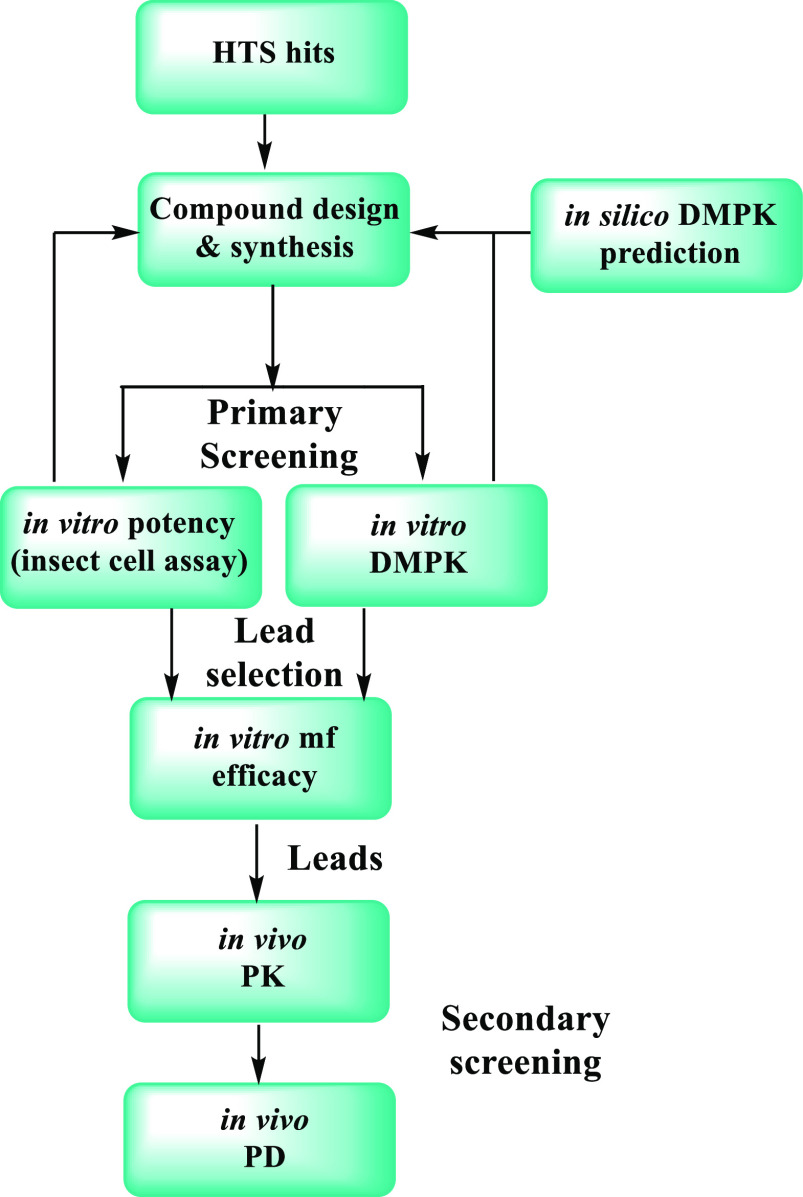
Screening cascade for the design, synthesis and testing of anti-*Wolbachia* compounds.

**Table 1:**
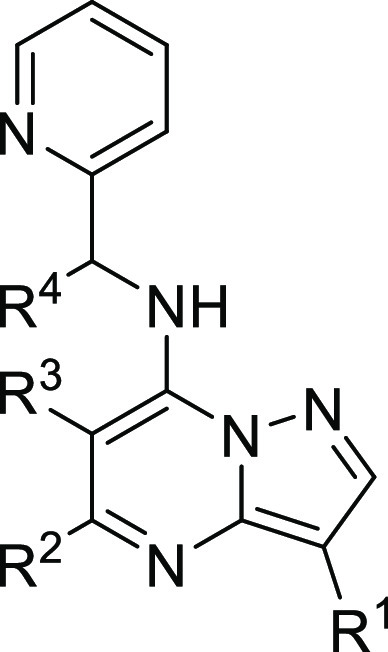

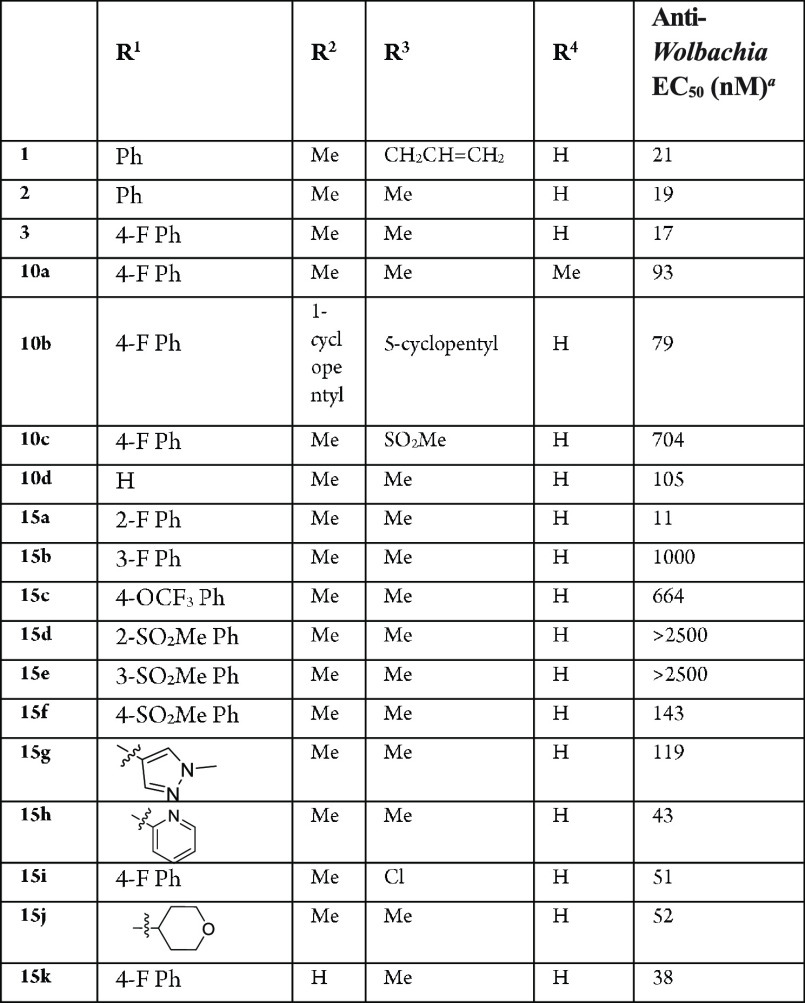
*In Vitro* Potency Data of Key Analogues

a Note: All tested compounds showed no cytotoxicity against the insect cells at the top concentration (5 μM) in the assay.

Despite the high potency (EC_50_ = 21 nM) of the original hit **1**, DMPK assessments highlighted poor metabolic stability and low aqueous solubility. (Figure 1) The phenyl ring, allyl substitution, and methylene linker were some of the positions within the hit molecule that were predicted to be susceptible to oxidative CYP metabolism. Hence, the early hit to lead optimization was focused on enhancing the metabolic stability while maintaining potency.

The initial synthetic route (Scheme 1) was developed to allow for the synthesis of analogues with modifications at the R^2^-, R^3^-, and 7-positions of the pyrazolopyrimidine ring. While many necessary 5-amino pyrazoles (**1a**, **5d**, R^1^ = Ph, H respectively) are commercially available, it was also possible to synthesize them in a two-step reaction from the corresponding nitrile (**4a**, R^1^ = 4-F-Ph) using base (NaOEt or LDA) and ethyl formate followed by hydrazine mediated cyclization. This was followed by an acid-catalyzed pyrimidine ring formation using the pyrazoles **1a**, **5a**,**d** and β-keto esters **1b**, **6a**–**f**.^20^

**Scheme 1:**
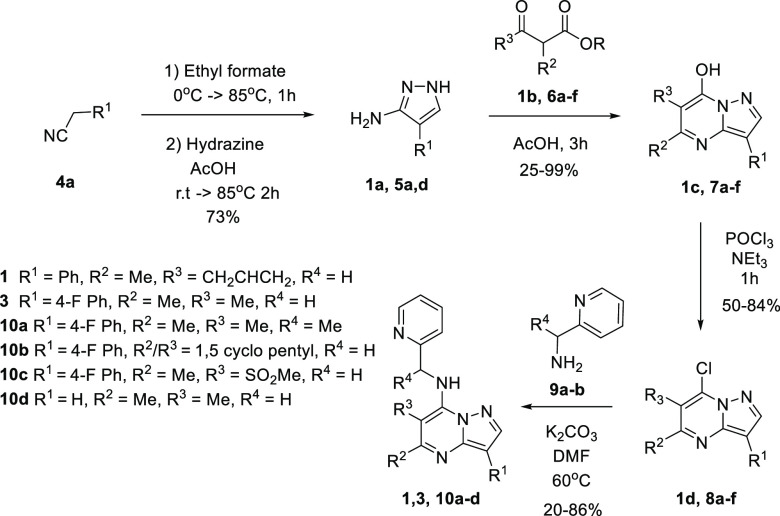
Synthetic Route for Analogues in the Pyrazolopyrimidine Template

When the 5-position of the pyrazolopyrimidine is unsubstituted (R^2^ = H), acid catalyzed cyclization is not suitable for the initial imine formation step. For these analogues, the pyrimidine ring was formed by reaction of the appropriate pyrazole and aldehyde to give the imine intermediate which cyclizes upon addition of KO^*t*^Bu. Chlorination of the 7-hydroxy pyrimidine intermediates **1c**, **7a**–**f** was achieved using phosphoryl chloride and subsequent substitution of intermediates **1d**, **8a**–**f** was achieved by aromatic substitution with the appropriate amines, **9a**,**b**, to produce the pyrazolopyrimidines, **1**, **3**, and **10a**–**d**.

While it is possible to use the appropriate nitriles as starting material for the synthesis of target compounds with different groups at the R^1^-position, a divergent orientated synthesis at this position was desirable for the production of a number of target analogues from a common intermediate. (Scheme 2) Functionalization at the R^1^-position can also be achieved *via* iodination of the pyrazolopyrimidines **8d**–**f** (R^1^ = H) using *N*-iodosuccinimide (NIS).^2^ This pathway requires Boc-protection of the free amines **12** to allow for Suzuki-mediated coupling of intermediates **13** with the corresponding boronic acid. Finally TFA-mediated Boc-deprotection of compounds **14a**–**k** afforded the desired targets **15a**–**k**, which contained a variety of monosubstituted phenyl rings and nitrogen heterocycles.

**Scheme 2:**
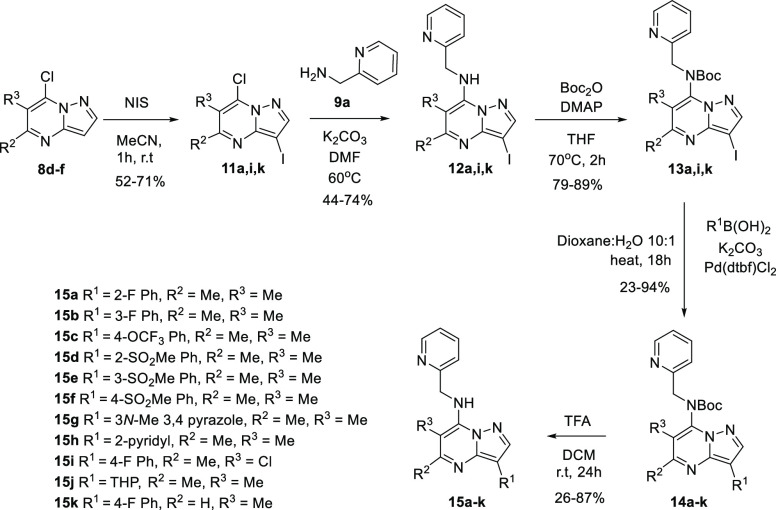
Optimized Route for the Synthesis of Analogues with Modified 3-Positions Note: THP = Tetrahydropyran.

## Substitutions at the R^3^-Position

Replacement of the allyl group at this position in the original hit **1** with a methyl substituent resulted in excellent potency (compound **2**, Table 1, EC_50_ = 19 nM) and improved metabolic stability as shown by rat hepatocyte clearance of compound **2** (Table 2). The analogue with ring-fusion of a lipophilic cyclopentyl ring at R^2^ and R^3^ (**10b**) showed good anti-*Wolbachia* activity (EC_50_ = 79 nM) and demonstrated that modifications at both positions could be tolerated. This modification did not improve aqueous solubility, but the cyclopentyl ring connecting these two positions increased metabolic stability. Chlorine was also tolerated at the R^3^-position in terms of potency and has a positive effect on metabolic stability in comparison to the corresponding 6-methyl analogues (**15i** vs **3**). On the other hand, SO_2_Me group at the R^3^-position (**10c**) reduced anti-*Wolbachia* activity noticeably.

**Table 2:** *In Vitro* DMPK Data of Pyrazolopyrimidine Analogues^a^

	LogD_7.4_	Aq. Sol. (μM)	H. Mics. CL.(μL/min/mg)	R. Hep. CL (μL/min/10^6^ cells)	H. PPB (%)
**1**	4.2	0.07	63.22	105.60	99.9
**2**	4.0	0.40	ND	59.03	99.80
**3**	4.3	0.90	49.19	67.66	99.8
**10a**	4.3	0.30	24.49	75.43	ND
**10b**	3.3	0.50	48.86	41.25	99.0
**10c**	3.7	0.01	8.76	46.48	99.7
**10d**	2.0	561	261.80	109.60	77.0
**15a**	4.0	0.60	83.97	113.20	99.9
**15b**	4.1	1.00	59.33	86.16	99.9
**15c**	4.5	5.00	8.76	156.60	97.9
**15d**	2.3	11.0	74.15	>300.00	88.0
**15e**	3.0	2.00	12.08	147.80	96.6
**15f**	3.3	2.00	21.78	27.58	99.8
**15g**	2.7	43.0	44.83	10.31	91.8
**15h**	2.3	30.0	243.60	144.90	97.4
**15i**	4.3	2.0	44.62	26.37	99.5
**15j**	2.4	21.0	23.65	71.84	ND
**15k**	4.0	6.0	37.48	45.53	99.6

a Notes: Aq. Sol. = aqueous solubility in pH7.4 PBS; H. Mics. CL = intrinsic human microsomal clearance measured *in vitro*; R. Hep. CL = intrinsic Rat hepatocyte clearance measured *in vitro*; ND = not determined.

## Substitutions at the R^4^-Position

In an effort to reduce the potential metabolism of the methylene linker, an analogue with a methyl substitution at this position (**10a**) was investigated. Methylation of the linker imparts a 2-fold increase in human microsomal stability, but this modification leads to a 4-fold loss in activity (compounds **10a** vs **3**). According to HTS results (data not shown here), we found that the SAR was restricted at the 2′-pyridyl side-chain. Any minor modifications to this ring significantly reduced compound potency. For this reason, we focused more on the R^1^-position of the pyrazolopyrimidine core since the HTS data suggested that a wider range of modifications might be tolerated at this position.

## Substitutions at the R^1^-Position

The R^1^-aryl side chain is not essential for anti-*Wolbachia* activity; however, removal of the aromatic ring resulted in some reduction in potency (**10d** EC_50_ = 105 nM) coupled with a significant increase in aqueous solubility. A range of small substitutions at the *para*-position of the phenyl ring are generally tolerated, while a 4-fluoro substitution (**3**) appears to be optimal for activity (EC_50_ = 17 nM). Substitution of the polar SO_2_Me group at the *para*-position (**15f**) resulted in reducing LogD, increasing aqueous solubility and improving metabolic stability. However, similar substitution with the SO_2_Me group at the *ortho-* and *meta*-positions of the phenyl ring is not tolerated, as shown by compounds **15d** and **15e**, where substitution results in a significant reduction in potency. On the other hand, smaller substituents, such as fluorine are tolerated in the *ortho-*position although these small substitutions offered no significant improvements to DMPK properties. While compound **3** displayed improved *in vitro* metabolic stability comparing with HTS hit **1**, it still suffered from poor aqueous solubility.

In an attempt to reduce LogD further and to improve aqueous solubility, analogues **15g** and **15h** containing the heterocyclic aromatic 1-methyl-1*H*-pyrazolyl and 2-pyridyl groups were synthesized as more polar/LogD reducing phenyl ring replacements. These modifications both greatly enhanced the aqueous solubility and lowered LogD with the 2-pyridyl analogue **15h** maintaining the majority of its potency.

Compound **15g** proved to be reasonably stable metabolically; however, a near 10-fold drop in activity supported further investigation into other areas of the scaffold to improve overall properties. Exploration of saturated heterocyclic ring systems led to the synthesis of compound **15j** containing the tetrahydropyran (THP) moiety. The THP side-chain is well tolerated for potency (EC_50_ = 52 nM) while improving DMPK properties comparing with HTS hit **1**.

## Substitutions at the R^2^-Position

Substitution at this position is not essential for anti-*Wolbachia* activity. It was demonstrated that the removal of the methyl substituent from the R^2^-position (**15k**) was tolerated in terms of potency and resulted in some improvement in DMPK properties when compared to compound **3**. This result suggests the R^2^-position was another area that could be further explored for optimization of potency and DMPK in future work.

## Anti-*Wolbachia* Activity Assessment in *B. malayi* mf Assay

The mf assay is an orthogonal *in vitro* assay that uses *B. malayi* microfilariae to confirm the anti-*Wolbachia* activity of tested compounds against the human parasitic nematode.^21,22^ After being screened for potency and DMPK properties *in vitro*, a number of selected analogues were tested at 5 μM alongside the gold-standard doxycycline for comparison of anti-*Wolbachia* activity in the mf assay. The majority of tested compounds demonstrated good activity, comparable to doxycycline, in this assay except for **15i** (Table 3). The secondary readout of the mf assay is the motility of the mf by the tested compounds comparing with vehicle control. For anti-*Wolbachia* drugs, such as doxycycline, they should not affect the motility of the parasitic worm since this indicates off-target effects. For this reason, the chloro-substituted analogue **15i** was considered unsuitable for further development as an anti-*Wolbachia* drug as it had significant effects on worm motility in the assay.

**Table 3:** *In Vitro* Potency of Key Analogues in the *B*. *malayi* mf Assay

molecule	anti-*Wolbachia* potency from cell assay EC_50_ (nM)	anti-*Wolbachia* potency from mf assay (6 days at 5 μM % *Wolbachia* reduction in wsp:gst^a^)
DOX	17	86.5
**1**	21	75.60
**3**	17	83.20
**10d**	105	77.10
**15f**	143	80.40
**15i**	51	toxic^b^
**15j**	52	85

a wsp, *Wolbachia* surface protein copy number, median % reduction cf. vehicle (DMSO) control; gst, GST copy number (single copy gene, worm size biomarker).

b Significantly reduced motility of the mf after 6 days incubation comparing with vehicle (DMSO) control.

## *In Vivo* Pharmacokinetic (PK) Profiling in Mice

**Chart 1:**
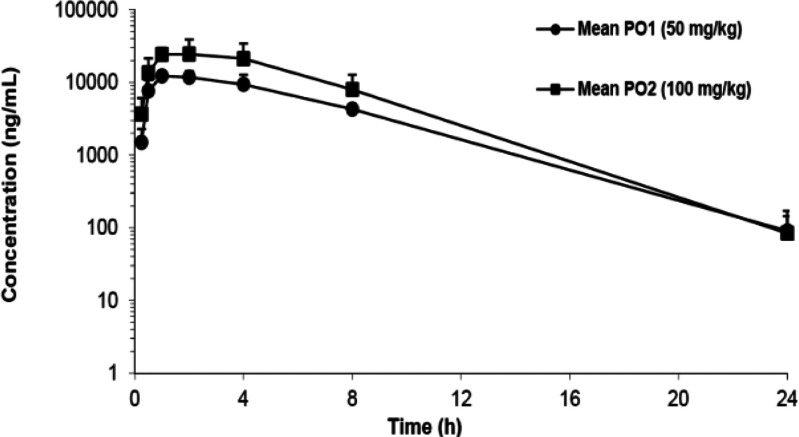
Mean Plasma Concentration of Compound **15f** Following Dosing to SCID Mice with SSV

Taking all the *in vitro* results into consideration, compounds **15f** and **15j** possessed a suitable balance of high potency, good DMPK properties and acceptable preliminary safety profiles (e.g., cytotoxicity and hERG inhibition^23^), and **15f** was chosen for *in vivo* PK evaluation. Compound **15f** was dosed orally to SCID mice at 50 and 100 mg/kg using a standard suspended vehicle (SSV); results from the study are shown in Chart 1 and Table 4. Despite limited aqueous solubility, this compound demonstrates good tolerability, excellent *in vivo* PK profiles with high exposure, reasonable half-life and dose-proportional AUC. Based on this data **15f** has been selected as a lead for an *in vivo* proof-of-concept pharmacodynamics study and further optimization.

**Table 4:** *In Vivo* PK Profile of Compound **15f**, Dosing to SCID Mice Using SSV

dosage (oral)	50 mg/kg	100 mg/kg
*T*_1/2_ (h)	2.9	2.4
*C*_max_ (μg/L)	13 067	27 800
*T*_max_ (h)	1.33	1.67
AUC_0–*t*_ (μg·h/L)	82 209	162 617

**Figure 3:**
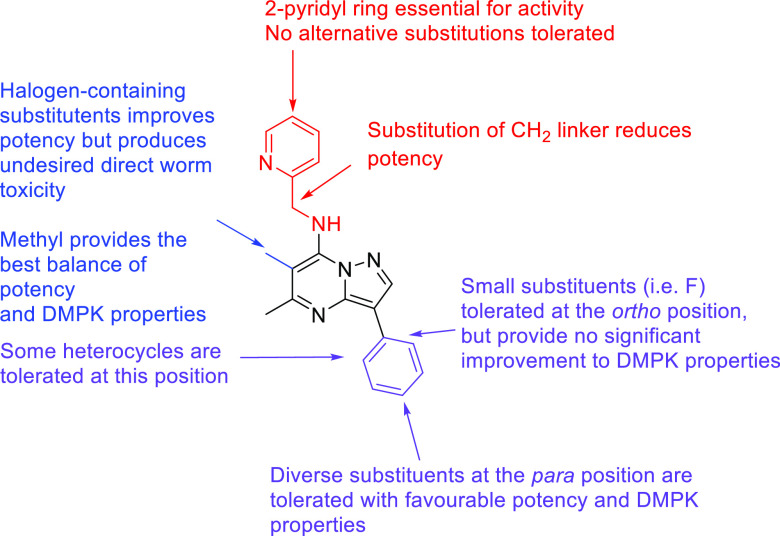
Summary of the SAR of anti-*Wolbachia* pyrazolopyrimidines.

In summary, the initial potent pyrazolopyrimidine hit **1**, which has poor metabolic stability and inadequate aqueous solubility parameters, has been optimized to provide a number of highly potent compounds with enhanced DMPK properties as represented by lead molecules **15f** and **15j**. A summary of the key SAR is highlighted in Figure 3.

More explorations of the R^1^-position and potential functionalization of the 2-position of the pyrazolopyrimidine core could further improve the anti-*Wolbachia* potency and the overall DMPK properties. These future directions will be determined by the results of *in vivo* efficacy studies of **15f** which will be reported in due course. In addition, the *in vivo* PK and PD studies of the other lead, **15j** will be triggered if the proof-of-concept *in vivo* efficacy study of **15f** is positive.

## Supporting Information Available

*In vitro* biological testing methods, experimental procedure of the synthesis, and characterization of the reported compounds (PDF)

## Abbreviations

LF: lymphatic filariasis
DEC: diethylcarbamazine
HTS: high-throughput screening
DMPK: drug metabolism pharmacokinetics
DOX: doxycycline
PD: Pharmacodynamics
PK: pharmacokinetics
SAR: structure–activity relationships
*B.malayi*: *Brugia malayi*
mf: microfilariae
SCID: severe combined immunodeficient
