# Supplementary material for: Development of Pyrazolopyrimidine Anti-Wolbachia Agents for the Treatment of Filariasis
Source: ACS Med Chem Lett. 2021 Aug 20;12(9):1421–6. doi: 10.1021/acsmedchemlett.1c00216 (PMC8436242; doi:10.1021/acsmedchemlett.1c00216)
Supplement: Supplementary file 1 — ml1c00216_si_001.pdf [file ml1c00216_si_001.pdf]

Supporting Information

Development of Pyrazolopyrimidine Anti-*Wolbachia* Agents for the Treatment of Filariasis

Paul McGillan,[ǂ] Neil G. Berry,[ǂ] Gemma L. Nixon,[ǂ] Suet C. Leung,[ǂ] Peter J. H. Webborn,[^] Mark C. Wenlock,[^] Stefan Kavanagh,[‖] Andrew Cassidy, [¥] Rachel H. Clare,[¥] Darren A. Cook,[¥] Kelly L. Johnston,[¥§] Louise Ford,[¥] Stephen A. Ward,[¥] Mark J. Taylor [¥] W. David Hong, [ǂ¥]* and Paul M. O'Neill [ǂ]*.

[ǂ] Department of Chemistry, University of Liverpool, Crown Street L69 7ZD, U.K.

[¥] Department of Tropical Disease Biology, Liverpool School of Tropical Medicine, Pembroke Place, Liverpool, L3 5QA, U.K.

[^] Drug Safety & Metabolism, IMED Biotech Unit, AstraZeneca UK, Cambridge, CB2 0AA, U.K.

[‖] Oncology Safety Sciences, Clinical Pharmacology & Safety Sciences, R&D, AstraZeneca, Cambridge, CB2 OAA, U.K.

[§] Institute of Systems, Molecular & Integrative Biology, School of Life Sciences, University of Liverpool, Crown Street L69 7ZB, U.K.

Table of contents

Synthetic methods, procedures and chemical analysis data S2

Biological testing methods and procedures S29

Synthetic methods, procedures and chemical analysis data

General

Air- and moisture-sensitive reactions were performed in oven dried glassware sealed with rubber septa under an atmosphere of nitrogen from a manifold or balloon. Anhydrous solutions and sensitive liquids were transferred via syringe or stainless steel cannula. Reactions were stirred using Teflon-coated magnetic stir bars. Organic solutions were concentrated using a Buchi rotary evaporator with a diaphragm vacuum pump.

Purification of solvents and reagents

Anhydrous solvents were either purchased from Sigma Aldrich or dried and distilled immediately prior to use under a constant flow of dry nitrogen. Tetrahydrofuran was distilled from Na, dichloromethane and Et_3N were distilled from CaH_2. All reagents were purchased from Sigma Aldrich, Alfa Aesar, Frontier Scientific, Apollo Scientific, Fluorochem and were used without any purification unless otherwise indicated. Prep-HPLC purification was carried out using Gilson-281. The HPLC conditions/method: a Phenomenex Synergi C18 (30 mm x 150 mm, 5 μm) column at 25°C and a mixture of eluent A) water + 0.225% formic acid and eluent B) acetonitrile.

Purification of products

Thin layer chromatography (TLC) was performed on 0.25 mm Merck silica gel 60 F254 plates and visualised by ultraviolet light. U.V. inactive compounds were visualised using iodine, p-anisaldehyde solution, ninhydrin or potassium permanganate followed by gentle heating. Flash column chromatography was performed using normal phase silica gel purchased from Sigma-Aldrich and an air line to apply pressure.

Analysis

Melting points were determined by a Gallenkamp apparatus and are uncorrected. ^{1}H NMR spectra were recorded on Bruker AMX 400 (400 MHz) spectrometer and reported as chemical shifts on parts per million (ppm, δ) relative to tetramethylsilane (TMS) as the internal reference, multiplicity (s = singlet, br s = broad singlet, d = doublet, t = triplet, q =quartet, m = multiplet), coupling constant (*J*, Hz), integration. ^{13}C NMR spectra were recorded on Bruker AMX400 (101 MHz) spectrometer and reported in terms of chemical shift (ppm, δ) relative to residual solvent peak. Mass spectra (MS) and high resolution mass spectra (HRMS) were recorded on a VG analytical 7070E machine, Fisons TRIO spectrometers using electron ionisation (EI) and chemical ionisation (CI), and Micromass LCT mass spectrometer using electron spray ionisation (ESI). All mass values are within error limits of ±5 ppm.

Elemental analyses (%C, %H, %N) were determined by the University of Liverpool Microanalysis Laboratory, reported percentages are within error limits of ±0.5 %. LCMS analysis was carried out using SHIMADZU LCMS-2020 with a Chromolith@Flash RP-18e (25 x 2.0 mm) column at 25°C and a mixture of eluent A) 0.0375% TFA in water (v/v) and eluent B) 0.01875% TFA in Acetonitrile (v/v) as mobile phase, with run time: 1.5 or 2 min.

*Compounds **2**, **5d**, **6a-b**, **6d-e** and **9a-b** are commercially available.

Synthesis of 4-(4-fluorophenyl)-1H-pyrazol-3-amine (5a)

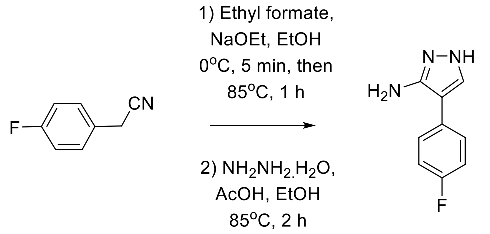

5a

To a mixture of NaOEt (6.04 g, 88.7 mmol) in ethanol (100 mL) was added 2-(4-fluorophenyl)acetonitrile (8.87 mL, 82.6 mmol) and the mixture was allowed to stir at 0°C for 5 min. Ethyl formate (14.88 mL, 185 mmol) was added to the reaction, which was then heated to 85°C and stirred for 1 h. Volatiles were removed under reduced pressure and the residue formed was diluted with water and 2M HCl was added to adjust to pH 3-4. The aqueous was extracted with ethyl acetate (x 3). Combined organic layer was washed with brine and dried over Na_2SO_4, filtered and evaporated to dryness. To crude 2-(4-fluorophenyl)-3-oxo-propane nitrile in ethanol (100 ml) was added $NH_2NH_2.H_2O$ (7.12 mL, 147.1 mmol) followed by AcOH (6 mL, 104.9 mmol). The reaction was allowed to stir at 85 °C for 2 h before cooling to room temperature and evaporating to dryness, to afford **5a** as yellow solid (73%): ^{1}H NMR (400 MHz, DMSO) δ 11.69 (s, 1H), 7.65 (s, 1H), 7.52 (dd, *J* = 8.6, 5.6 Hz, 2H), 7.15 (t, *J* = 8.9 Hz, 2H), 4.73 (s, 2H) ; HRMS (CI) $C_9H_9N_3F$ $[M+H]^+$ requires 178.0780, found 178.0772.

Synthesis of methyl 2-acetylpent-4-enoate (1b)

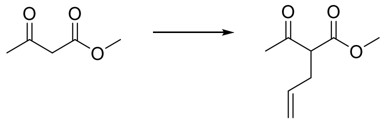

1b

To methyl acetoacetate (5.4 ml, 50 mmol) in THF (15 mL) at 0°C was added NaH (1.32 g, 55 mmol) and the reaction was allowed to stir for 30 min. Allyl bromide (6.6 g, 55 mmol) was then added to the reaction mixture before allowing the reaction to warm to room temperature and stirring overnight. The reaction was evaporated to dryness, the residue was diluted with ethyl acetate and washed with water. The organic layer was dried over $MgSO_4$, filtered and evaporated to dryness to give the crude product. The crude was purified by column chromatography eluting with 20% ethyl acetate in hexane to afford **1b** as colourless oil (63%): ^{1}H NMR (400 MHz, $CDCl_3$) δ 5.86 – 5.64 (m, 1H), 5.10 – 5.06 (m, 2H), 3.74 (s, 3H), 3.55 (t, *J* = 7.4 Hz, 1H), 2.60 (t, *J* = 8.2 Hz, 2H), 2.24 (s, 3H). MS (ES) $C_8H_{13}O_3$ $[M+H]^+$ requires 157.09, found 157.1 (rel intensity 100).

Synthesis of ethyl 2-(methylsulfonyl)-3-oxobutanoate (6c)

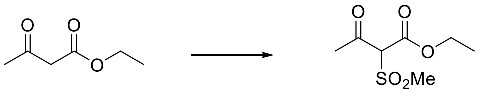

6c

To a mixture of ethyl 3-oxobutanoate (2.0 g, 15.4 mmol) in THF (20 mL) was added NaH (922.08 mg, 23.1 mmol, 1.5 eq) in portions at 0 ℃ under N_2 protection. The mixture was allowed to stir at 0 ℃ for 30 min. Then methanesulfonyl chloride (3.89 g, 30.7 mmol, 2 eq) was added and the mixture was warmed to 25 ℃ and stirred for 2 h (followed by TLC). The mixture was diluted with ethyl acetate (60 mL) and washed with saturated brine (40 mL x 2), dried with Na_2SO_4, filtered and concentrated *in vacuo*. The residue was purified by column chromatography eluting with 5-15% ethyl acetate in petroleum ether to afford **6c** as a yellow liquid (1.00 g, crude), which was used directly in the next step without further purification.

Synthesis of ethyl 2-methyl-3-oxopropanoate (6f)

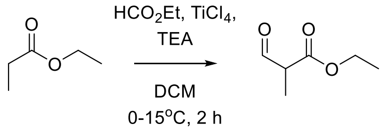

6f

A solution of ethyl propanoate (25.0 g, 244.8 mmol) and ethyl formate (36.27 g, 489.6 mmol, 2 eq) in DCM (200 mL) at 0 ℃ was added $TiCl_4$ (92.86 g, 489.4 mmol, 2 eq) followed by triethylamine (59.45 g, 587.5 mmol, 2.4 eq) dropwise under N_2 protection. The reaction mixture was allowed to stir at 0 ℃ for 1 h and at 10-15 ℃ for another 1 h (followed by TLC). The reaction mixture was poured into ice water and extracted with DCM (100 mL x 3). The organic layer was washed with brine (100 mL x 3), dried over Na_2SO_4, filtered and concentrated to give **6f** as a brown oil (50.0 g, crude), which was used directly for next step without further purification.

General Procedure 1 - Synthesis of pyrazolo[1,5-a]pyrimidin-7-ol (1c, 7a-f)

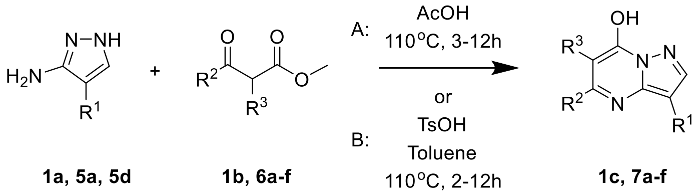

1a, 5a, 5d **1b, 6a-f** **1c, 7a-f**

(A) To the corresponding 3-amino-1,2-pyrazole (5 mmol) in AcOH (8 ml) was added the appropriate β-keto ester (20 mmol, 4 eq) and the reaction was allowed to stir at 110°C for 3-12 h (followed by TLC). The reaction was cooled to room temperature and diluted with Et_2O causing precipitation of the desired product which was filtered and washed with further Et_2O. The product was dried under high vacuum and was used without further purification.

(B) To the corresponding 3-amino-1,2-pyrazole (5 mmol) in toluene (10 ml) was added the appropriate β-keto ester (5 mmol, 1 eq) and TsOH (0.1 eq). The reaction was allowed to stir at 110°C for 2-12 h (followed by TLC). The reaction was cooled to room temperature and filtered and washed with petroleum ether. The filtrate collected was concentrated *in vacuo* to give the desired product. The product was dried under high vacuum and was used without further purification.

Synthesis of 6-allyl-5-methyl-3-phenylpyrazolo[1,5-a]pyrimidin-7-ol (1c)

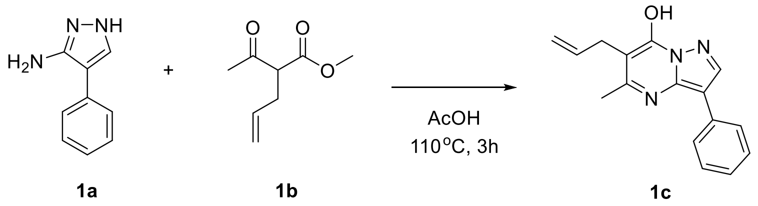

3-amino-4-phenyl-1H-pyrazole (**1a**) and **1b** were treated according to **General Procedure 1A** to give **1c** as a solid (88%): ^{1}H NMR (400 MHz, DMSO) δ 11.75 (s, 1H), 8.11 (s, 1H), 7.57 (d, *J* = 7.7 Hz, 2H), 7.46 (t, *J* = 7.7 Hz, 2H), 7.32 (t, *J* = 7.4 Hz, 1H), 5.86 (m, 1H), 5.02 (m, 2H), 3.27 (d, *J* = 5.9 Hz, 2H), 2.38 (s, 3H); MS (ES) $C_{16}H_{16}N_3O$ $[M+H]^+$ requires 266.13, found 266.1 (rel intensity 100).

Synthesis of 3-(4-fluorophenyl)-5,6-dimethylpyrazolo[1,5-a]pyrimidin-7-ol (7a)

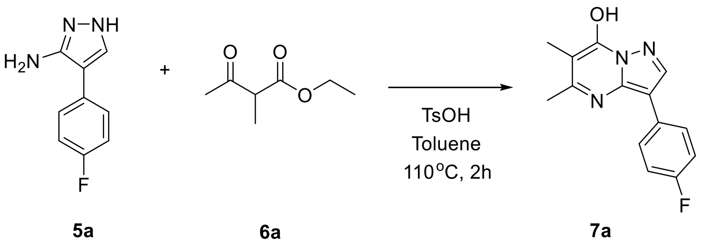

5a and ethyl 2-methylacetoacetate (**6a**) were treated according to **General Procedure 1B** to give **7a** as a white solid (81%): ^{1}H NMR (400 MHz, DMSO) δ 11.65 (s, 1H), 8.06 (s, 1H), 7.59 (dd, *J* = 8.7, 5.5 Hz, 2H), 7.29 (t, *J* = 8.9 Hz, 2H), 2.37 (s, 3H), 2.00 (s, 3H); HRMS (CI) $C_{14}H_{13}N_3OF$ $[M+H]^+$ requires 258.1043, found 258.1035.

Synthesis of 3-(4-fluorophenyl)-6,7-dihydro-5H-cyclopenta[d]pyrazolo[1,5-a]pyrimidin-8-ol (7b)

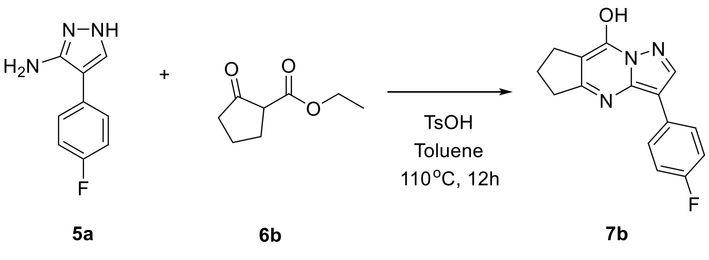

5a and ethyl 2-oxocylopentancarboxylate (**6b)** were treated according to **General Procedure 1B** to give **7b** as a grey solid (76%): ^{1}H NMR (300 MHz, DMSO) δ 12.26 (br. s, 1H), 8.10 (s, 1H), 7.61 – 7.47 (m, 2H), 7.38 – 7.04 (m, 3H), 2.95 (t, *J* = 7.6 Hz, 3H), 2.71 (t, *J* = 7.2 Hz, 3H), 2.22 – 1.80 (m, 2H).

Synthesis of 3-(4-fluorophenyl)-5-methyl-6-(methylsulfonyl)pyrazolo[1,5-a]pyrimidin-7-ol (7c)

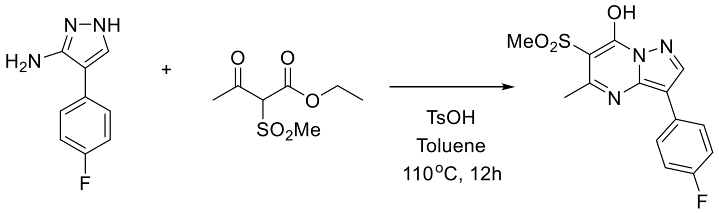

5a 6c 7c

5a and **6c** were treated according to **General Procedure 1B** to give **7c** as a yellow solid (25%): ^{1}H NMR (400 MHz, DMSO) δ 12.60 (br. s, 1H), 8.22 (s, 1H), 7.82 – 7.47 (m, 2H), 7.32 (t, *J* = 8.8 Hz, 2H), 3.33 (s, 3H), 2.73 (s, 3H); LCMS (Method 10-80AB): R_t = 0.702 min / 2 min, 89%, MS (ES) $C_{14}H_{13}N_3O_3FS$ $[M+H]^+$ requires 322.07, found 332.1.

Synthesis of 5,6-dimethylpyrazolo[1,5-a]pyrimidin-7-ol (7d)

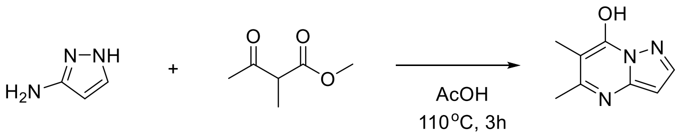

5d 6d 7d

3-Aminopyrazole (**5d**) and methyl 2-methyl-3-oxobutanoate (**6d**) were treated according to **General Procedure 1A** to give **7d** as a white solid (99%): ^{1}H NMR (400 MHz, DMSO) δ 12.11 (s, 1H), 7.80 (d, *J* = 2.0 Hz, 1H), 6.04 (d, *J* = 2.0 Hz, 1H), 2.30 (s, 3H), 1.96 (s, 3H); HRMS (CI) $C_8H_{10}N_3O$ $[M+H]^+$ requires 164.0818, found 164.0821.

Synthesis of 6-chloro-5-methylpyrazolo[1,5-a]pyrimidin-7-ol (7e)

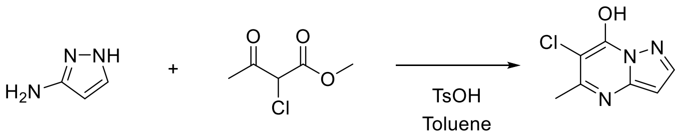

5d 6e 7e

3-Aminopyrazole (**5d**) and methyl 2-chloro-3-oxobutanoate (**6e**) were treated according to **General Procedure 1B** to give **7e** as an off-white solid (91%): ^{1}H NMR (400 MHz, DMSO) δ 12.81 (br. s, 1H), 7.90 (d, *J* = 2.0 Hz, 1H), 6.17 (d, *J* = 2.0 Hz, 1H), 2.44 (s, 3H).

Synthesis of 6-methylpyrazolo[1,5-a]pyrimidin-7-ol (7f)

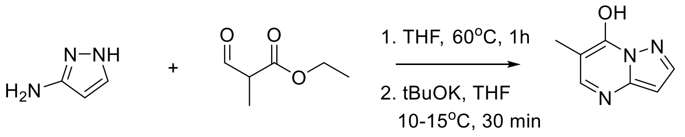

To a mixture of **5d** (17.9 g, 215.2 mmol) and **6f** (35.0 g, 268.9 mmol, 1.25 eq) in THF (300 mL) was allowed to stir at 60 ℃ for 1 h (followed by TLC). The reaction mixture was used directly for next step without further work up and purification.

To a mixture of ethyl 3-((1H-pyrazol-3-yl)imino)-2-methylpropanoate (52.0 g, 266.4 mmol, 1 eq) in THF (300 mL) obtained from last step at 0 ℃ was added ^{t}BuOK (29.9 g, 266.4 mmol, 1 eq) in portions and the mixture was allowed to stir at 10-15 ℃ for 30 min. Solids were precipitated (followed by TLC). The mixture was cooled to 0 ℃ and acified with HCl/EtOAc (4 M) to pH7. The mixture was concentrated to give **7f** as a light yellow solid (60.0 g, crude) contained KCl. And it was used directly for next step without further purification.

General Procedure 2 - Synthesis of 7-chloropyrazolo[1,5-a]pyrimidines (1d, 8a-f)

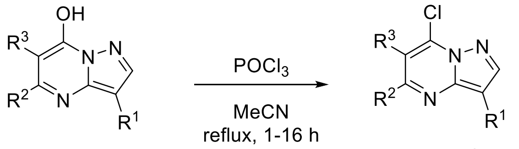

To a solution of pyrazolo[1,5-a]pyrimidin-7-ol (5 mmol) in acetonitrile (10 mL) was added $POCl_3$ (4 eq). The reaction mixture was allowed to stir at reflux temperature for 1-16 h (followed by TLC). The mixture was cooled to room temperature and poured into ice-water slowly. The mixture was basified with sat. $NaHCO_3$ (aq) to pH 7, The mixture was extracted with ethyl acetate (x 3), the combined organic layer was washed with water, followed by brine, dried over $MgSO_4$, filtered and concentrated *in vacuo*. The crude was then purified by column chromatography using 10-50% ethyl acetate in hexane to give the desired product.

Synthesis of 6-allyl-7-chloro-5-methyl-3-phenylpyrazolo[1,5-a]pyrimidine (1d)

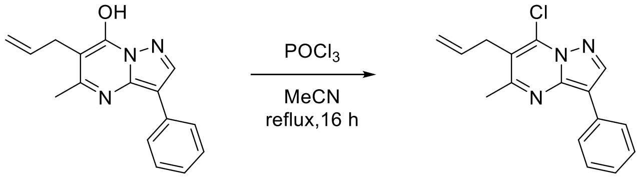

1c was treated according to **General Procedure 2** to give **1d** as a yellow solid (84%):^{1}H NMR (400 MHz, DMSO) δ 8.81 (s, 1H), 8.20 (d, *J* = 8.3 Hz, 2H), 7.50 (t, *J* = 7.8 Hz, 2H), 7.31 (t, *J* = 7.4 Hz, 1H), 6.03 (ddt, *J* = 17.1, 10.2, 5.6 Hz, 1H), 5.19 (dd, *J* = 10.2, 1.5 Hz, 1H), 5.09 (dd, *J* = 17.2, 1.6 Hz, 1H), 3.66 (d, *J* = 5.6 Hz, 2H), 2.69 (s, 3H); MS (ES) $C_{16}H_{15}N_3Cl$

[M+H]$^+$ requires 284.10 and 286.10, found 284.1 (rel intensity 100) and 286.1 (rel intensity 27).

Synthesis of 7-chloro-3-(4-fluorophenyl)-5,6-dimethyl pyrazolo[1,5-a]pyrimidine (8a)

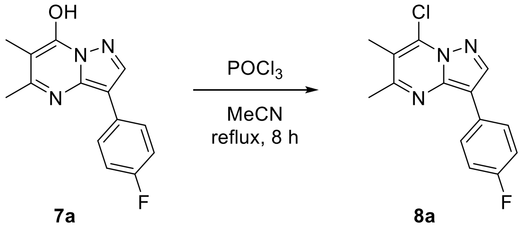

7a was treated according to **General Procedure 2** to give **8a** as a yellow solid (50%): ^{1}H NMR (400 MHz, $CDCl_3$) δ 8.36 (s, 1H), 8.14 – 7.91 (m, 2H), 7.21 – 7.05 (m, 2H), 2.67 (s, 3H), 2.46 (s, 3H); HRMS (ES) $C_{14}H_{11}N_3F^{35}Cl$ [M+H]$^+$ requires 276.0704, found 276.0700, $C_{14}H_{11}N_3F^{37}Cl$ [M+H]$^+$ requires 278.0674, found 278.0681.

Synthesis of 7-chloro-3-(4-fluorophenyl)-6,7-dihydro-5H-cyclopenta[d]pyrazolo[1,5-a]pyrimidine (8b)

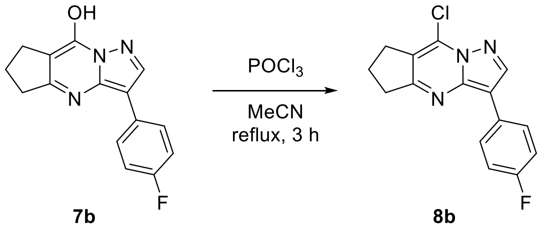

7b was treated according to **General Procedure 2** to give **8b** as a light green solid (81%): ^{1}H NMR (400 MHz, DMSO) δ 8.71 (s, 1H), 8.21 – 8.03 (m, 2H), 7.33 – 7.19 (m, 2H), 3.09 (t, *J* = 7.7 Hz, 2H), 3.02 (t, *J* = 7.4 Hz, 2H), 2.20 (p, *J* = 7.6 Hz, 2H).

Synthesis of 7-chloro-3-(4-fluorophenyl)-5-methyl-6-(methylsulfonyl)pyrazolo[1,5-a]pyrimidine (8c)

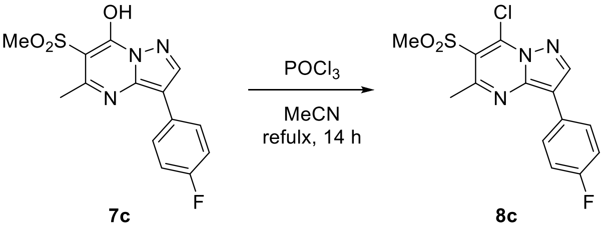

7c was treated according to **General Procedure 2** to give **8c** as a brown solid, which was used directly in the next step without any purification.

Synthesis of 7-chloro-5,6-dimethylpyrazolo[1,5-a]pyrimidine (8d)

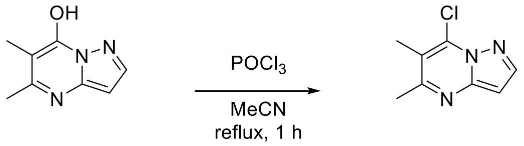

7d **8d**

7d was treated according to **General Procedure 2** to give **8d** as a white solid (81%): ^{1}H NMR (400 MHz, $CDCl_3$) δ 8.09 (d, *J* = 2.3 Hz, 1H), 6.63 (d, *J* = 2.3 Hz, 1H), 2.61 (s, 3H), 2.43 (s, 3H); HRMS (CI) $C_8H_9N_3{}^{35}Cl$ $[M+H]^+$ requires 182.0480, found 182.0488.

Synthesis of 6,7-dichloro-5-methylpyrazolo[1,5-a]pyrimidine (8e)

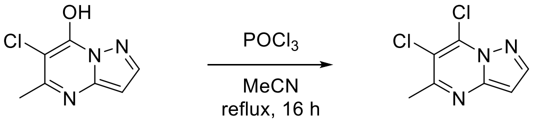

7e **8e**

7e was treated according to **General Procedure 2** to give **8e** as a brown oil (60%): ^{1}H NMR (400 MHz, $CDCl_3$) δ 8.16 (d, *J* = 2.3 Hz, 1H), 6.70 (d, *J* = 2.3 Hz, 1H), 2.73 (s, 3H).

Synthesis of 7-chloro-6-methylpyrazolo[1,5-a]pyrimidine (8f)

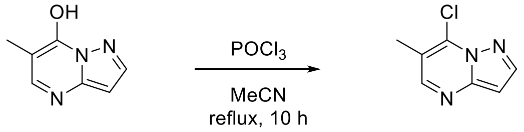

7f **8f**

7f was treated according to **General Procedure 2** to give **8f** as a brown solid (62%), which was used directly in the next step without any purification.

LCMS (Method 0-60AB): R_t = 1.323 min / 2 min, 35%, MS (ES) $C_7H_7N_3Cl$ $[M+H]^+$ requires 168.03, found 168.1.

Synthesis of 7-chloro-3-iodo-5,6-dimethylpyrazolo[1,5-a]pyrimidine (11a)

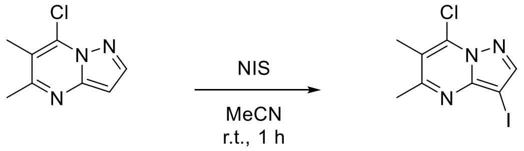

8d **11a**

8d (463 mg, 2.6 mmol) and NIS (573 mg, 2.6 mmol) in anhydrous MeCN (25 mL) were allowed to stir at room temperature for 1 h (followed by TLC). Volatiles were removed *in vacuo* and the residue was diluted with DCM (20 mL). The solution was washed with water

(10 mL) and brine (10 mL). The organic layer was dried over $MgSO_4$, filtered and evaporated to dryness. The crude was purified by column chromatography using dichloromethane to isolate **11a** as a white solid (71%): ^{1}H NMR (400 MHz, $CDCl_3$) δ 8.11 (s, 1H), 2.67 (s, 3H), 2.45 (s, 3H); HRMS (CI) $C_8H_8N_3ICl$ $[M+H]^+$ requires 307.9446, found 307.9450.

Synthesis of 6,7-dichloro-3-iodo-5-methylpyrazolo[1,5-a]pyrimidine (11i)

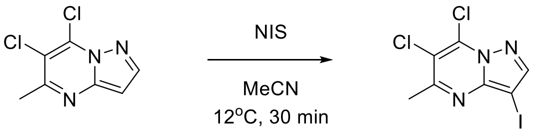

8e **11i**

To a solution of **8e** (1 g, 5 mmol) in CH_3CN (3 mL) was added NIS (1.1 g, 5 mmol, 1 eq). The mixture was allowed to stir at 12 ℃ for 30 min (followed by TLC). The mixture was concentrated *in vacuo*. The residue was purified by column chromatography eluting with 5-15% ethyl acetate in petroleum ether to give **11i** as a brown solid (900 mg, 55% yield): ^{1}H NMR (400 MHz, $CDCl_3$) δ 8.19 (s, 1H), 2.81 (s, 3H).

Synthesis of 7-Chloro-3-iodo-6-methylpyrazolo[1,5-a]pyrimidine (11k)

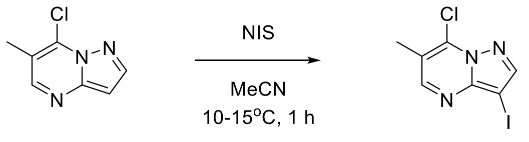

8f **11k**

A mixture of **8f** (6.50 g, 38.8 mmol) and NIS (8.73 g, 38.8 mmol, 1 eq) in CH_3CN (60 mL) was allowed to stir at 10-15 ℃ for 1 h (followed by TLC). The mixture was concentrated to give a residue. The residue was purified by column chromatography using 5-15% ethyl acetate in petroleum ether to give **11k** as a brown solid (6.0 g, 52% yield): ^{1}H NMR (400 MHz, $CDCl_3$) δ 8.43 (s, 1H), 8.21 – 8.15 (m, 1H), 2.49 (s, 3H).

General Procedure 3 - the substitution of 7-chloro-pyrazolo[1,5-a]pyrimidines (1, 3, 10a-d, 12a, 12i, 12k)

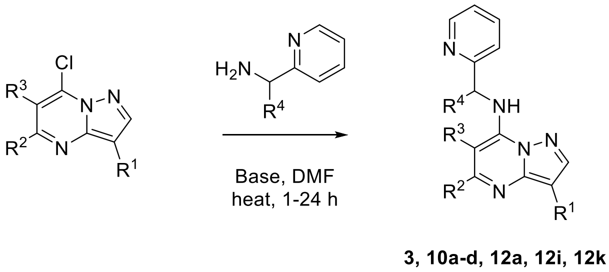

3, 10a-d, 12a, 12i, 12k

(A) To a solution of 7-chloropyrazolo[1,5-a]pyrimidine (1 mmol) in anhydrous DMF (5 mL) was added 2-picolylamine (1.2 eq) and triethylamine (2 eq). The reaction mixture was allowed to stir at 60-110°C for 1-4 h (followed by TLC). Water (5 mL) was slowly added

to the mixture. Precipitates formed were collected by filtration. If no precipitate was formed, the mixture was extracted with ethyl acetate, the organic layer was washed with brine, dried over Na_2SO_4, filtered and concentrated. The crude solid/residue was then purified by column chromatography or prep-HPLC to give the desired product.

(B) To a solution of 7-chloropyrazolo[1,5-a]pyrimidine (1 mmol) in anhydrous DMF (5 mL) was added 2-picolylamine (1 eq) and K_2CO_3 (2 eq). The reaction mixture was allowed to stir at 60°C/reflux for 2 – 24 h (followed by TLC). The solvent was removed *in vacuo* and the residue was then extracted with ethyl acetate, washed with water, followed by brine, dried over Na_2SO_4, filtered and concentrated. The crude was then purified by column chromatography or prep-HPLC to give the desired product.

Synthesis of 6-allyl-5-methyl-3-phenyl-*N*-(pyridin-2-ylmethyl)pyrazolo[1,5-a]pyrimidin-7-amine (1)

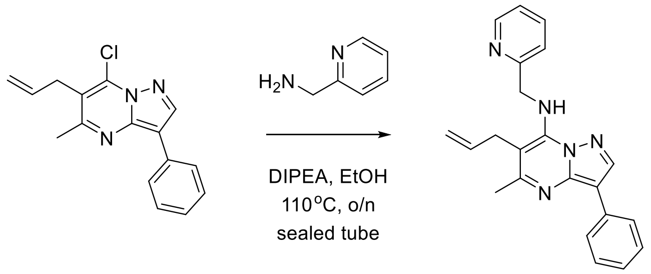

1d **1**

To a solution of **1d** (0.24 g, 0.85 mmol) in anhydrous ethanol (10 mL) in a sealed tube was added 2-picolylamine (0.85 mmol, 1 eq) followed by DIPEA (1.02 mmol, 1.2 eq). The resulting solution was allowed to stir at 110°C in the sealed tube overnight (followed by TLC). The solvent was evaporated *in vacuo* and the residue was purified by column chromatography using 40-80% ethyl acetate in hexane to give **1** as an off white solid (75%): Melting point: 158-160°C; ^{1}H NMR (400 MHz, DMSO) δ 8.63 – 8.52 (m, 2H), 8.16 (d, *J* = 7.7 Hz, 2H), 8.02 (t, *J* = 5.9 Hz, 1H), 7.79 (t, *J* = 7.2 Hz, 1H), 7.43 – 7.35 (m, 3H), 7.33 – 7.27 (m, 1H), 7.16 (t, *J* = 7.3 Hz, 1H), 6.28 – 5.87 (m, 1H), 5.17 – 5.08 (m, 3H), 4.94 (d, *J* = 16.9 Hz, 1H), 3.51 (d, *J* = 4.2 Hz, 2H), 2.48 (s, 3H); HRMS (ES) $C_{22}H_{22}N_5$ $[M+H]^+$ requires 356.1875, found 356.1879; Anal. $C_{22}H_{21}N_5$ requires C 74.34%, H 5.96%, N 19.70%, found C 74.10%, H 5.92%, N 19.40%.

Synthesis of 3-(4-fluorophenyl)-5,6-dimethyl-*N*-(pyridin-2-ylmethyl)pyrazolo[1,5-a]pyrimidin-7-amine (3)

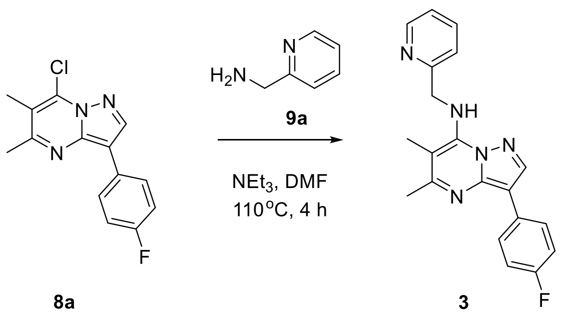

8a **3**

8a and 2-picolylamine (**9a**) were treated according to **General Procedure 3A**, the crude product was purified by column chromatography eluting with 50% ethyl acetate in hexane to give **3** as an off-white solid (86%): Melting point: 146-148°C; ^{1}H NMR (400 MHz, DMSO) δ 8.72 (d, *J* = 5.1 Hz, 1H), 8.51 (br. s, 1H), 8.39 (s, 1H), 8.23 (t, *J* = 7.6 Hz, 1H), 8.03 – 7.92 (m, 2H), 7.80 (d, *J* = 8.1 Hz, 1H), 7.73 – 7.58 (m, 1H), 7.25 (t, *J* = 8.9 Hz, 2H), 5.51 (s, 2H), 2.55 (s, 3H), 2.29 (s, 3H); HRMS (ES) $C_{20}H_{19}N_5F$ requires 348.1624, found 348.1620. ; Anal $C_{20}H_{18}N_5F$ requires C 69.15%, H 5.22%, N 20.16%, found C 69.50%, H 5.34%, N 19.80%; LCMS (Method 5-95AB): R_t = 0.724 min / 1.5 min, 100%, MS (ES): m/z $[M+H]^+$ 348.0.

Synthesis of 3-(4-Fluorophenyl)-5,6-dimethyl-*N*-(1-(pyridin-2-yl)ethyl)pyrazolo[1,5-a]pyrimidin-7-amine (10a)

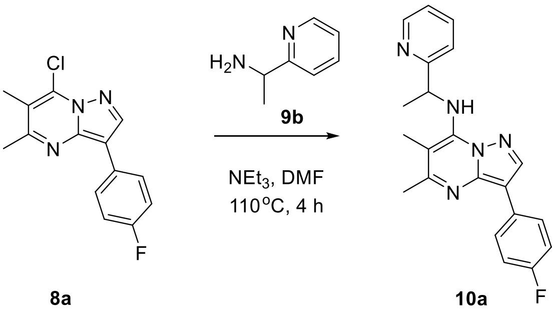

8a and 1-(2-pyridyl)ethylamine (**9b**) were treated according to **General Procedure 3A** to give **10a** as a light yellow solid (52%): ^{1}H NMR (400 MHz, DMSO) δ 8.73 (d, *J* = 4.8 Hz, 1H), 8.47 (s, 1H), 8.25 – 8.15 (m, 1H), 8.08 – 7.96 (m, 2H), 7.88 (d, *J* = 7.8 Hz, 1H), 7.71 – 7.61 (m, 1H), 7.25 (t, *J* = 8.9 Hz, 2H), 6.23 (br. s, 1H), 2.55 (s, 3H), 2.31 (s, 3H), 1.71 (d, *J* = 6.8 Hz, 3H); LCMS (Method 5-95AB): R_t = 0.787 min / 1.5 min, 99%, MS (ES) $C_{21}H_{21}N_5F$ $[M+H]^+$ requires 362.18, found 362.0.

Synthesis of 3-(4-Fluorophenyl)-*N*-(pyridin-2-ylmethyl)-6,7-dihydro-5H-cyclopenta[d] pyrazolo[1,5-a]pyrimidin-8-amine (10b)

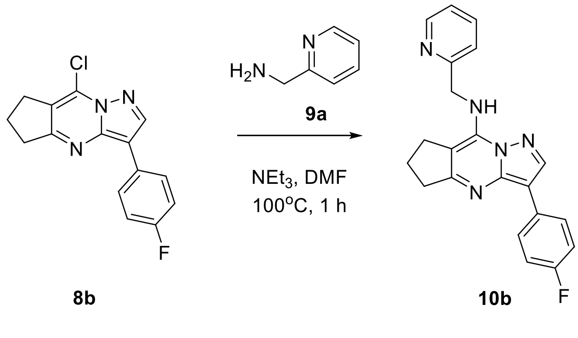

8b and 2-picolylamine (**9a**) were treated according to **General Procedure 3A** to give **10b** as a white solid (42%): ^{1}H NMR (400 MHz, DMSO) δ 8.59 – 8.53 (m, 2H), 8.33 (t, *J* = 6.4 Hz, 1H), 8.22 – 8.11 (m, 2H), 7.80 (td, *J* = 7.7, 1.7 Hz, 1H), 7.37 (d, *J* = 7.9 Hz, 1H), 7.35 – 7.29 (m, 1H), 7.22 (t, *J* = 9.0 Hz, 2H), 4.96 (d, *J* = 6.5 Hz, 2H), 3.02 (t, *J* = 7.3 Hz, 2H), 2.85 (t, *J* = 7.8 Hz, 2H), 2.04 – 1.92 (m, 2H); LCMS (Method 10-80AB): R_t = 1.554min / 2 min, 95%, MS (ES) $C_{21}H_{19}N_5F$ $[M+H]^+$ requires 360.16, found 360.1.

Synthesis of 3-(4-fluorophenyl)-5-methyl-6-(methylsulfonyl)-*N*-(pyridin-2-ylmethyl) pyrazolo[1,5-a]pyrimidin-7-amine (10c)

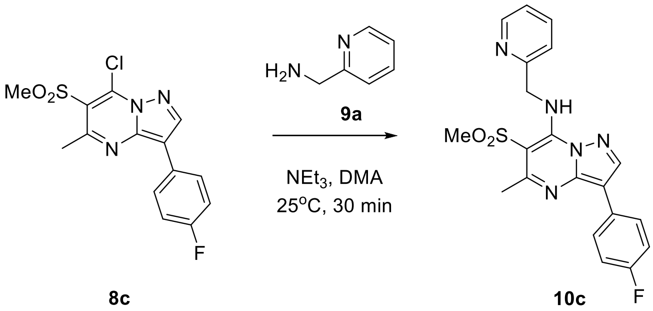

8c **10c**

To mixture of **8c** (200 mg, 0.59 mmol) and 2-picolylamine (**9a**) (82.75 mg, 0.76 mmol, 1.3 eq) in DMA (5 mL) was added excess Et_3N (3 ml) in one portion under N_2. The mixture was allowed to stir at 25 ℃ for 30 min (followed by TLC). The mixture was poured into water (20 mL). The mixture was diluted with ethyl acetate (30 mL), washed with saturated brine (10 mL x 4), dried over Na_2SO_4, filtered and concentrated *in vacuo*. The residue was purified by prep-TLC using 25% ethyl acetate in petroleum ether followed by trituration with dichloromethane (2 mL) to afford **10c** as light yellow solid (48 mg, 20% yield): ^{1}H NMR (400 MHz, DMSO) δ 9.63 (t, *J* = 5.3 Hz, 1H), 8.67 (s, 1H), 8.52 (d, *J* = 4.2 Hz, 1H), 8.21 – 8.06 (m, 2H), 7.82 (td, *J* = 7.7, 1.7 Hz, 1H), 7.46 (d, *J* = 7.9 Hz, 1H), 7.31 (dd, *J* = 6.7, 5.1 Hz, 1H), 7.28 – 7.21 (m, 2H), 5.66 (d, *J* = 5.3 Hz, 2H), 3.41 (s, 3H), 2.76 (s, 3H); LCMS (Method 5-95AB): R_t = 0.846 min / 1.5 min, 94%, MS (ES) $C_{20}H_{19}N_5O_2FS$ $[M+H]^+$ requires 412.12, found 412.0.

Synthesis of 5,6-dimethyl-*N*-(pyridin-2-ylmethyl)pyrazolo[1,5-a]pyrimidin-7-amine (10d)

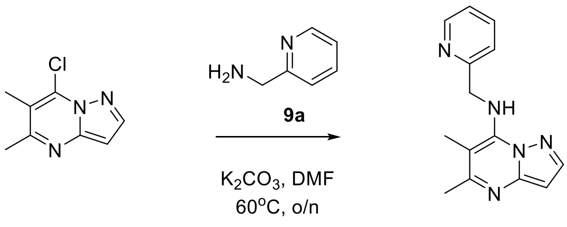

8d **10d**

8d and 2-picolylamine (**9a**) were treated according to **General Procedure 3B** to give **10d** as a white solid (68%): ^{1}H NMR (400MHz, DMSO) δ 8.56 (d, *J* = 4.7 Hz, 1H), 7.97 (d, *J* = 2.2 Hz, 1H), 7.81 – 7.74 (m, 2H), 7.37 (d, *J* = 7.9 Hz, 1H), 7.29 (dd, , *J* = 7.1, 5.2 Hz 1H), 6.27 (d, *J* = 2.2 Hz, 1H), 5.16 (d, *J* = 6.1 Hz, 2H), 2.41 (s, 3H), 2.27 (s, 3H); HRMS (ES) $C_{14}H_{16}N_5$ $[M+H]^+$ requires 254.1406, found 254.1401; Anal. $C_{14}H_{15}N_5$ requires C 66.38%, H 5.97%, N 27.65%, found C 66.14%, H 5.91%, N 27.81%.

Synthesis of 3-iodo-5,6-dimethyl-*N*-(pyridin-2-ylmethyl)pyrazolo[1,5-a]pyrimidin-7-amine (12a)

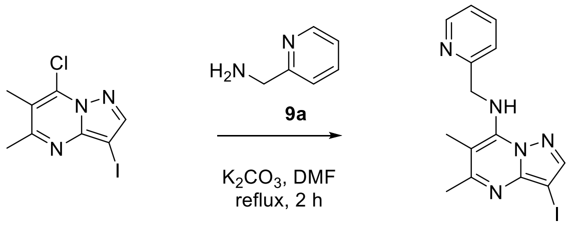

11a **12a**

11a and 2-picolylamine (**9a**) were treated according to **General Procedure 3B** to give **12a** as a pale yellow solid (64%): ^{1}H NMR (400 MHz, DMSO) δ 8.53 (d, *J* = 4.4 Hz, 1H), 8.28 (s, 1H), 8.09 (t, *J* = 6.0, 1H), 7.76 (td, *J* = 7.7, 1.5 Hz, 1H), 7.37 (d, *J* = 7.8 Hz, 1H), 7.28 (dd, *J* = 6.8, 5.3 Hz, 1H), 5.27 (d, *J* = 6.0, 2H), 2.42 (s, 3H), 2.26 (s, 3H); HRMS (CI) $C_{14}H_{15}N_5{}^{127}I$ $[M+H]^+$ requires 380.0372, found 380.0365.

Synthesis of 6-Chloro-3-iodo-5-methyl-*N*-(pyridin-2-ylmethyl)pyrazolo[1,5-a]pyrimidin-7-amine (12i)

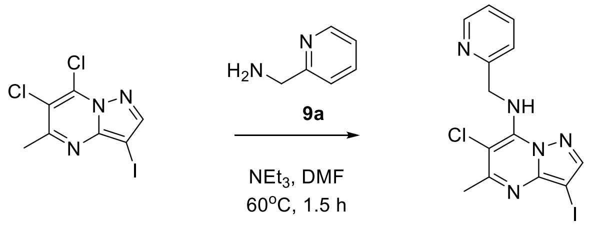

11i **12i**

11i and 2-picolylamine (**9a**) were treated according to **General Procedure 3A** to give **12i** as a grey solid (74%): ^{1}H NMR (400 MHz, DMSO) δ 8.51 (d, *J* = 4.1 Hz, 1H), 8.40 (t, *J* = 6.2 Hz, 1H), 8.14 (s, 1H), 7.77 (td, *J* = 7.7, 1.8 Hz, 1H), 7.35 (d, *J* = 7.9 Hz, 1H), 7.28 (dd, *J* = 6.9, 5.4 Hz, 1H), 5.35 (d, *J* = 6.1 Hz, 2H), 2.52 (s, 3H).

Synthesis of 3-Iodo-6-methyl-*N*-(pyridin-2-ylmethyl)pyrazolo[1,5-a]pyrimidin-7-amine (12k)

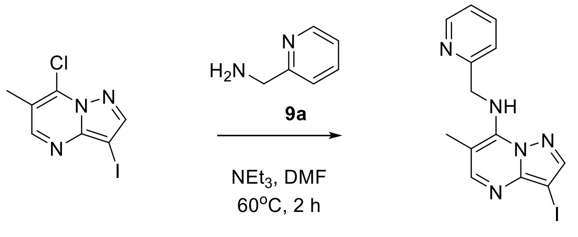

11k **12k**

12k and 2-picolylamine (**9a**) were treated according to **General Procedure 3A** to give **12k** as a light yellow solid (44%): ^{1}H NMR (400 MHz, $CDCl_3$) δ 8.59 (d, *J* = 4.8 Hz, 1H), 8.06 (s,

1H), 7.95 (s, 1H), 7.70 – 7.58 (m, 2H), 7.29 – 7.12 (m, 2H), 5.08 (d, *J* = 5.5 Hz, 2H), 2.40 (s, 3H).

Synthesis of *tert*-butyl (3-iodo-5,6-dimethylpyrazolo[1,5-a]pyrimidin-7-yl)(pyridin-2-ylmethyl)carbamate (13a)

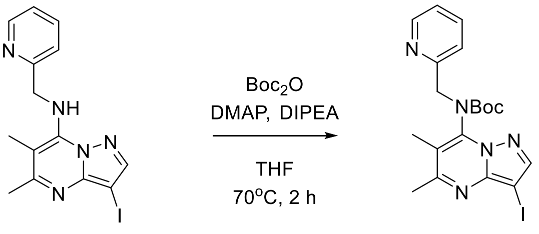

To a solution of **12a** (1.13 g, 2.97 mmol), DIPEA (1.24 ml, 5.95 mmol, 2 eq), and DMAP (0.182 g, 1.49 mmol, 0.5 eq) in dry THF (20 mL) was added Boc_2O (1.30 g, 5.95 mmol, 2 eq) and the reaction was allowed to stir at 70°C under nitrogen for 2 h. Upon completion of the reaction volatiles were removed under reduced pressure and the crude product was purified by column chromatography using 60% ethyl acetate in hexane to isolate the desired product **13a** as a white solid (79%): ^{1}H NMR (400 MHz, DMSO) δ 8.43 (d, *J* = 4.3 Hz, 1H), 8.27 (s, 1H), 7.79 (t, *J* = 7.6 Hz, 1H), 7.48 (d, *J* = 7.7 Hz, 1H), 7.31 (dd, *J* = 5.2, 1.9 Hz, 1H), 5.60 (d, *J* = 15.0 Hz, 1H), 4.71 (d, *J* = 15.0 Hz, 1H), 2.58 (s, 3H), 2.05 (s, 3H), 1.29 (s, 9H); HRMS (ES) $C_{19}H_{23}N_5O_2I$ $[M+H]^+$ requires 480.0897, found 480.0892.

Synthesis of *tert*-butyl (6-chloro-3-iodo-5-methylpyrazolo[1,5-a]pyrimidin-7-yl)(pyridin-2-ylmethyl)carbamate (13i)

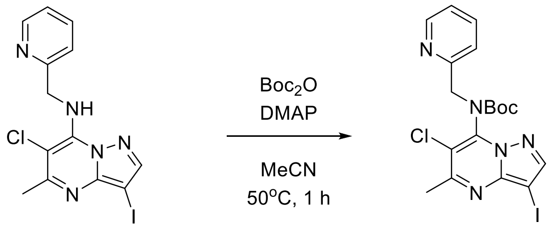

To a solution of **12i** (900 mg, 2.3 mmol) in CH_3CN (20 mL) was added Boc_2O (590 mg, 2.7 mmol, 1.2 eq) and DMAP (28 mg, 0.23 mmol, 0.1 eq). The mixture was allowed to stir at 50 ℃ for 1 h (followed by TLC). The mixture was concentrated. The residue was purified by column chromatographyeluting with 10-35% ethyl acetate in petroleum ether to give **13i** as a light brown solid (1 g, 89% yield): ^{1}H NMR (400 MHz, $CDCl_3$) δ 8.30 (d, *J* = 4.8 Hz, 1H), 8.04 (s, 1H), 7.77 – 7.51 (m, 2H), 7.12 (t, *J* = 5.1 Hz, 1H), 5.14 (d, *J* = 15.3 Hz, 1H), 5.01 (d, *J* = 15.3 Hz, 1H), 2.70 (s, 3H), 1.32 (s, 9H).

Synthesis of *tert*-butyl (3-iodo-6-methylpyrazolo[1,5-a]pyrimidin-7-yl)(pyridin-2-ylmethyl)carbamate (13k)

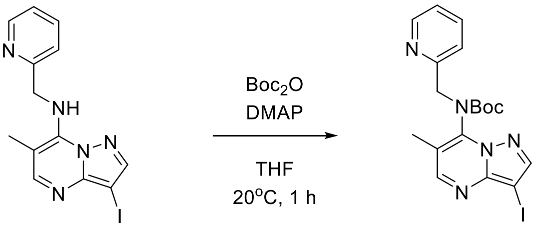

12k 13k

A mixture of **12k** (1.10 g, 3.01 mmol), Boc_2O (1.31 g, 6.02 mmol, 2 eq) and DMAP (36.8 mg, 0.3 mmol, 0.1 eq) in THF (30 mL) was allowed to stir at 15-20 ℃ for 1 h (followed by TLC). The mixture was concentrated to give a residue. The residue was purified by column chromatography using 10-50% ethyl acetate in petroleum ether to give **13k** as a light brown solid (1.20 g, 81% yield): ^1H NMR (400 MHz, $CDCl_3$) δ 8.39 (s, 1H), 8.09 (s, 1H), 7.64 – 7.56 (m, 1H), 7.45 (d, J = 7.8 Hz, 1H), 7.18 – 7.10 (m, 2H), 5.26 (d, J = 15.0 Hz, 1H), 4.77 (d, J = 14.9 Hz, 1H), 2.08 (s, 3H), 1.31 (s, 9H); LCMS (Method 10-80AB): R_t = 1.467 min / 2 min, 95%, MS (ES) $C_{18}H_{21}N_5O_2I$ $[M+H]^+$ requires 466.07, found 466.1.

General Procedure 4 – Suzuki reaction (14a-g)

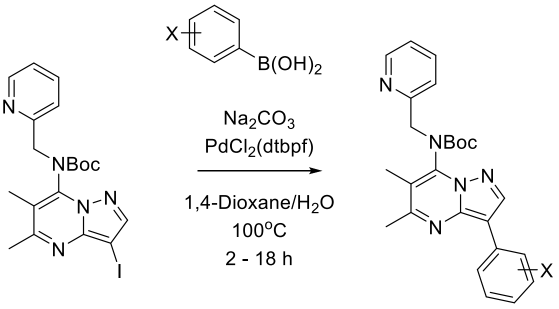

13a 14a-g

A suspension of 3-iodopyrazolo[1,5-a]pyrimidine (0.5 mmol), corresponding boronic acid (0.6 mmol, 1.2 eq), 1,1′-Bis(di-*tert*-butylphosphino)ferrocene]dichloropalladium(II) (0.05 mmol, 0.1 eq), Na_2CO_3 (1 mmol, 2 eq) in 1,4-dioxane (10 mL) and water (2 mL) was allowed to stir at 100 ℃ under N_2 for 2 – 18 h (followed by TLC). The reaction mixture was cooled to room temperature and the solvent was removed *in vacuo* to give a residue. The residue was purified by column chromatography or prep-HPLC or prep-TLC to give the desired product.

Synthesis of *tert*-butyl (3-(2-fluorophenyl)-5,6-dimethylpyrazolo[1,5-a]pyrimidin-7-yl)(pyridin-2-ylmethyl)carbamate (14a)

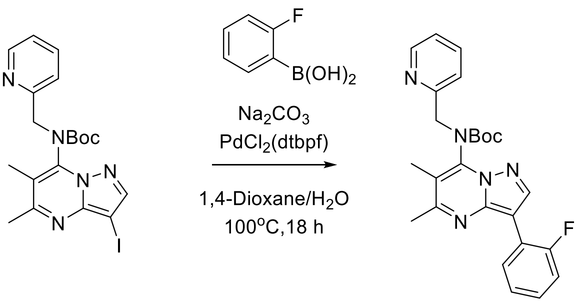

13a **14a**

13a and 2-fluorophenylboronic acid were treated according to **General Procedure 4** to give **14a** as a light yellow oil (79%): ^{1}H NMR (400 MHz, DMSO) δ 8.49 (m, 2H), 8.41 (d, *J* = 4.6 Hz 1H), 7.75 (t, *J* = 7.7 Hz, 1H), 7.47 (d, *J* = 7.8 Hz, 1H), 7.31 (m, 3H), 7.27 (dd, *J* = 7.1, 5.1 Hz, 1H), 5.18 (d, *J* = 15.1 Hz, 1H), 4.72 (d, *J* = 15.1 Hz, 1H), 2.58 (s, 3H), 2.04 (s, 3H), 1.26 (s, 9H); HRMS (ES) $C_{25}H_{27}N_5O_2F$ $[M+H]^+$ requires 448.2149, found 448.2148.

Synthesis of *tert*-butyl (3-(3-fluorophenyl)-5,6-dimethylpyrazolo[1,5-a]pyrimidin-7-yl)(pyridin-2-ylmethyl)carbamate (14b)

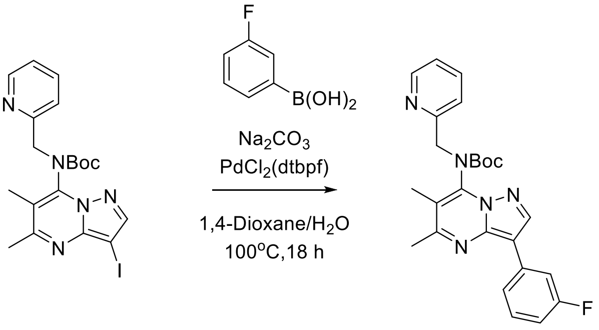

13a **14b**

13a and 3-fluorophenylboronic acid were treated according to **General Procedure 4** to give **14b** as a light yellow oil (81%), which was used in the next step without further purification.

Synthesis of *tert*-butyl (5,6-dimethyl-3-(4-(trifluoromethoxy)phenyl)pyrazolo[1,5-a]pyrimidin-7-yl)(pyridin-2-ylmethyl)carbamate (14c)

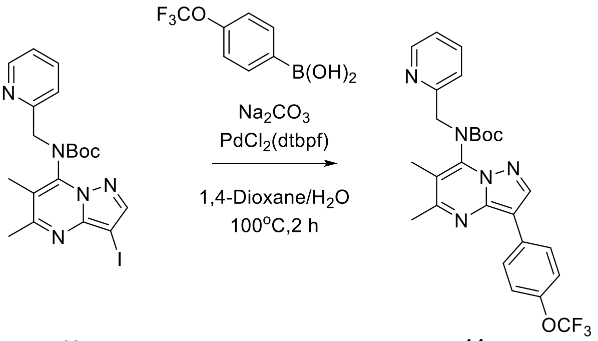

13a **14c**

13a and 4-(trifluoromethoxy)phenylboronic acid were treated according to **General Procedure 4** to give **14c** as a pale yellow oil (62%), which was used in the next step without further purification.

Synthesis of *tert*-butyl (5,6-dimethyl-3-(2-(methylsulfonyl)phenyl)pyrazolo[1,5-a]pyrimidin-7-yl)(pyridin-2-ylmethyl)carbamate (14d)

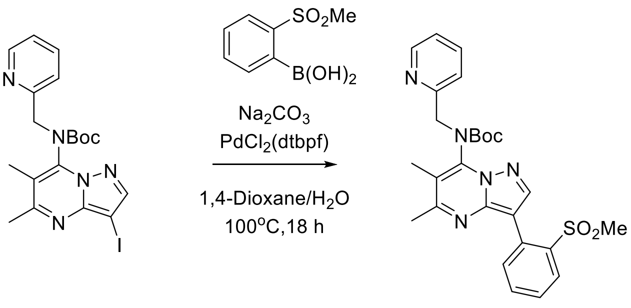

13a **14d**

13a and 2-(methylsulfonyl)phenylboronic acid were treated according to **General Procedure 4** to give **14d** as a light yellow oil (23%): ^{1}H NMR (400 MHz, MeOD) δ 8.24 (d, *J* = 4.6 Hz, 1H), 8.16 (s, 1H), 8.12 – 8.07 (m, 1H), 7.77 – 7.60 (m, 2H), 7.53 (m, 3H), 7.19 (dd, *J* = 6.8, 5.1 Hz, 1H), 5.11 (d, *J* = 14.9Hz, *1*H), 4.72 (d, *J* = 14.9 Hz, 1H), 2.7 (s, 3H), 2.38 (s, 3H), 1.91 (s, 3H), 1.22 (s, 9H); HRMS (ES) $C_{26}H_{29}N_5O_4S^{23}Na$ $[M+Na]^+$ requires 530.1838, found 530.1838.

Synthesis of *tert*-butyl (5,6-dimethyl-3-(3-(methylsulfonyl)phenyl)pyrazolo[1,5-a]pyrimidin-7-yl)(pyridin-2-ylmethyl)carbamate (14e)

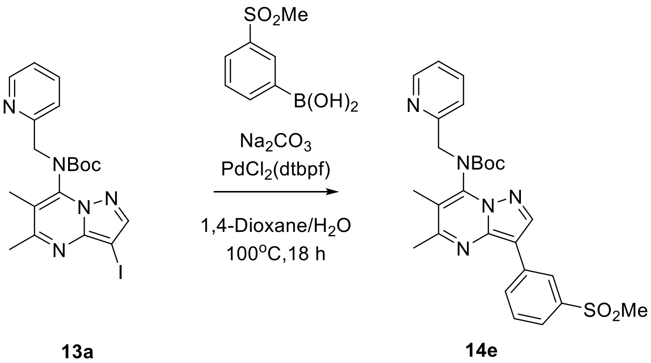

13a **14e**

13a and 3-(methylsulfonyl)phenylboronic acid were treated according to **General Procedure 4** to give **14e** as a light yellow oil (94%): ^{1}H NMR (400 MHz, DMSO) δ 8.88 (s, 1H), 8.77 (m, 1H), 8.58 (d, *J* = 7.6, 1.1 Hz, 1H), 8.45 (d, *J* = 4.1 Hz, 1H), 7.88 – 7.76 (m, 3H), 7.52 (d, *J* = 7.8 Hz, 1H), 7.32 (dd, *J* = 7.5, 5.0 Hz, 1H), 5.21 (d, *J* = 15.0 Hz, 1H), 4.78 (d, *J* = 15.1 Hz, 1H), 3.34 (s, 3H) 2.66 (s, 3H), 2.10 (s, 3H), 1.30 (s, 9H); HRMS (ES) $C_{26}H_{29}N_5O_4S^{23}Na$ $[M+Na]^+$ requires 530.1838, found 530.1845.

Synthesis of *tert*-butyl (5,6-dimethyl-3-(4-(methylsulfonyl)phenyl)pyrazolo[1,5-a] pyrimidin-7-yl)(pyridin-2-ylmethyl)carbamate (14f)

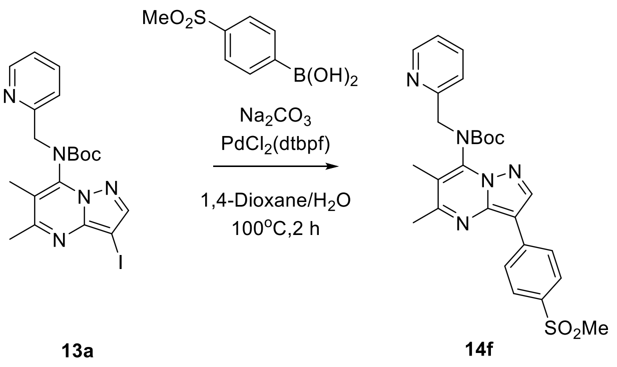

13a and 4-(methylsulfonyl)phenylboronic acid were treated according to **General Procedure 4** to give **14f** as a light yellow oil (62%), which was used in the next step without further purification.

Synthesis of *tert*-butyl (6-methyl-3-(1-methyl-1H-pyrazol-4-yl)pyrazolo[1,5-a]pyrimidin-7-yl)(pyridin-2-ylmethyl)carbamate (14g).

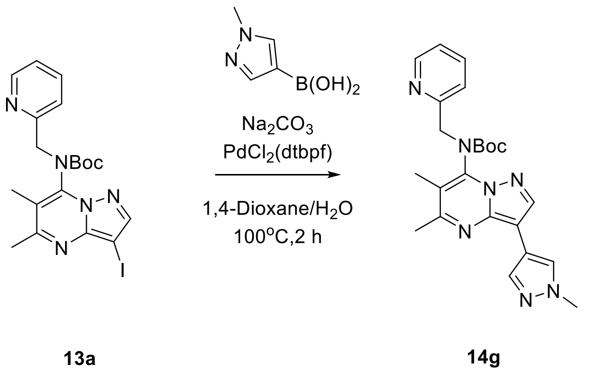

13a and 1-methyl-1H-pyrazole-4-boronic acid (1.25 eq) were treated according to **General Procedure 4** to give **14g** as a pale yellow oil (52%), which was used in the next step without further purification.

Synthesis of *tert*-butyl (5,6-dimethyl-3-(pyridin-2-yl)pyrazolo[1,5-a]pyrimidin-7-yl)(pyridin-2-ylmethyl)carbamate (14h)

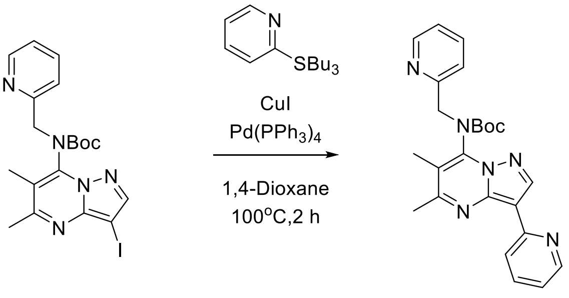

13a **14h**

To a mixture of **13a** (200 mg, 0.42 mmol), 2-(tributylstannyl)pyridine (215 mg, 0.58 mmol, 1.4 eq) and CuI (16 mg, 0.08 mmol, 0.2 eq) in anhydrous 1,4-dioxane (5 mL) was added Pd(PPh_3)$_4$ (48.2 mg, 0.04 mmol, 0.1 eq). The mixture was de-gassed and refilled with N_2, then heated at 100 ℃ for 2 h (followed by TLC). The mixture was added ethyl acetate (40 mL) and washed with a solution of KF (10 %, 20 mL). The organic layer was washed with brine (20 mL), dired over Na_2SO_4, filtered and concentrated *in vacuo* to give a residue, which was purified by column chromatography using 50% ethyl acetate in petroleum ether to give the crude product (purity: 80 %). The crude was further purified by prep-HPLC to give **14h** as a yellow solid (80 mg, 44%): LCMS (Method 5-95AB): R_t = 0.679 min / 1.5 min, 98%, MS (ES) $C_{24}H_{27}N_6O_2$ [M+H]$^+$ requires 431.22, found 431.2.

Synthesis of *tert*-butyl (6-chloro-3-(4-fluorophenyl)-5-methylpyrazolo[1,5-a]pyrimidin-7-yl)(pyridin-2-ylmethyl)carbamate (14i)

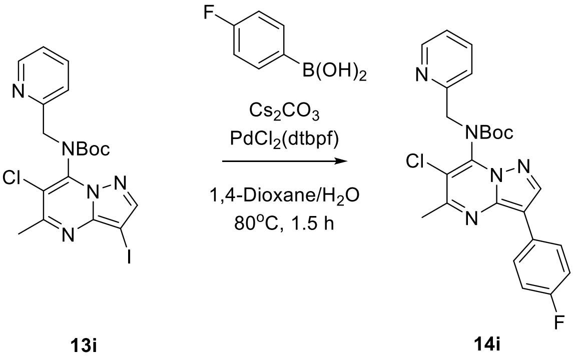

13i **14i**

To a solution of **13i** (150 mg, 0.3 mmol) and (4-fluorophenyl)boronic acid (63 mg, 0.45 mmol, 1.5 eq) in 1,4-dioxane (5 mL) and H_2O (1 mL) was added Cs_2CO_3 (195 mg, 0.6 mmol, 2 eq), 1,1′-Bis(di-*tert*-butylphosphino)ferrocene]dichloropalladium(II) (19 mg, 0.03 mmol, 0.1 eq). The mixture was allowed to stir at 80 ℃ for 1.5 h under N_2 (followed by TLC). The mixture was concentrated *in vacuo*. The residue was purified by column chromatography using 10-30% ethyl acetate in petroleum ether to give **14i** as a yellow solid (100 mg, 71%), which was used driectly in the next step without further purification.

Synthesis of *tert*-butyl (3-(3,6-dihydro-2H-pyran-4-yl)-5,6-dimethylpyrazolo[1,5-a]pyrimidin-7-yl)(pyridin-2-ylmethyl)carbamate (14j)

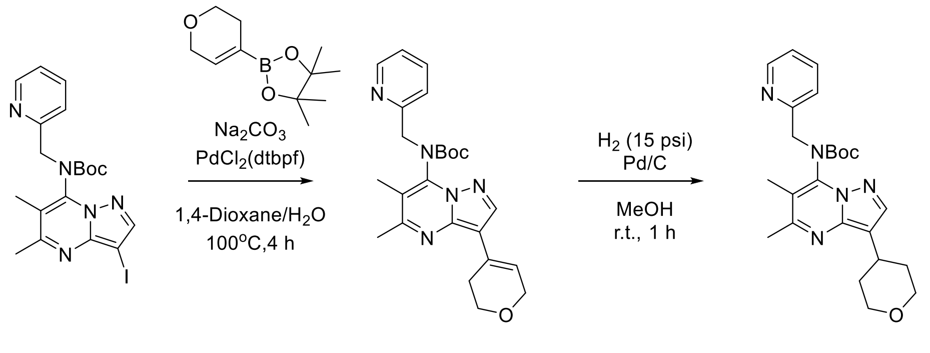

13a **14j_int** **14j**

A mixture of **13a** (200 mg, 0.42 mmol), 3,6-dihydro-2*H*-pyran-4-boronic acid pinacol ester (131.5 mg, 0.63 mmol, 1.5 eq), 1,1′-Bis(di-*tert*-butylphosphino)ferrocene] dichloropalladium(II) (27.2 mg, 0.04 mmol, 0.1 eq) and Na_2CO_3 (88.5 mg, 0.83 mmol, 2 eq) in 1,4-dioxane (20 mL) and H_2O (4 mL) was allowed to stir at 100 ℃ for 4 h under N_2 protection (followed by TLC). The mixture was concentrated to give a residue. The residue was purified by prep-TLC using 50% ethyl acetate in petroleum ether to give **14j_int** (100 mg, 55%) as a yellow solid.

A mixture of **14j_int** (100 mg, 0.23 mmol) and 10% Pd/C (10 mg) in MeOH (3 mL) was allowed to stir at room temperature for 1 h under H_2 (15 psi) (followed by TLC). The mixture was filtered through celite and the filtrate was concentrated *in vacuo* to give a residue. The residue was purified by prep-TLC using 50% ethyl acetate in petroleum ether to give **14j** as a colourless oil (100 mg, crude), which was used directly in the next step without further purification.

Synthesis of *tert*-butyl (3-(4-fluorophenyl)-6-methylpyrazolo[1,5-a]pyrimidin-7-yl)(pyridin-2-ylmethyl)carbamate (14k)

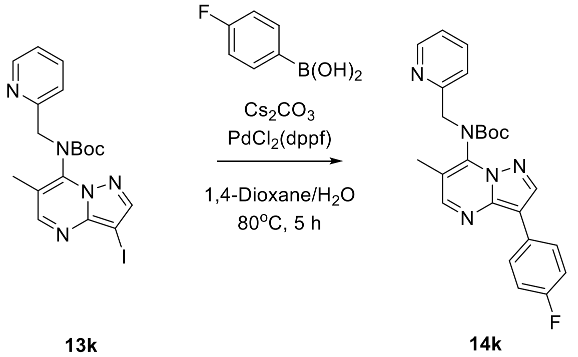

13k **14k**

A mixture of **13k** (100 mg, 0.21 mmol), (4-fluorophenyl)boronic acid (36.1 mg, 0.26 mmol, 1.2 eq), $PdCl_2$(dppf) (31.5 mg, 0.04 mmol, 0.2 eq) and Cs_2CO_3 (140 mg, 0.43 mmol, 2 eq) in 1,4-dioxane (3 mL) and H_2O (0.5 mL) was allowed to stir at 80 ℃ for 5 h under N_2 protection (followed by TLC). The mixture was concentrated *in vacuo* to give a residue. The residue was purified by prep-TLC using 50% ethyl acetate in petroleum ether to **14k** as a yellow solid (70 mg, 75%), which was used directly in the next step without further purification.

General Procedure 5 – Boc deprotection (15a-k)

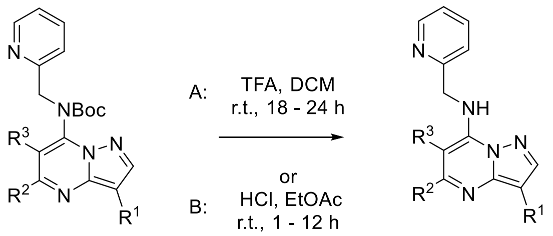

14a-k **15a-k**

(A) To the Boc-protected amine (1 mmol) in dichloromethane (8 mL) was added trifluoroacetic acid (2 mL). The reaction mixture was allowed to stir at room temperature overnight (followed by TLC). The solvent was removed *in vacuo*, the residue was diluted with ethyl acetate, washed with sat. $NaHCO_3$ (aq) (x 2), followed by brine, then dried over $MgSO_4$, filtered and concentrated. The crude was purified by column chromatography to give the desired product.

(B) To the Boc-protected amine (0.2 mmol) in ethyl acetate (2 mL) was added 4M HCl in ethyl acetate (4 mL), the resulting solution was allowed to stir at room temperature for 1 – 12 h (followed by TLC). The mixture was filtered and the filter cake was further washed with ethyl acetate (2 mL). The solid collected was dissolved in methanol (5 mL), and the solution was basified with strong base anion exchange resin and concentrated *in vacuo*. The residue was then purified by prep-HPLC or prep-TLC to give the desired product.

Synthesis of 3-(2-fluorophenyl)-5,6-dimethyl-N-(pyridin-2-ylmethyl)pyrazolo[1,5-a] pyrimidin-7-amine (15a)

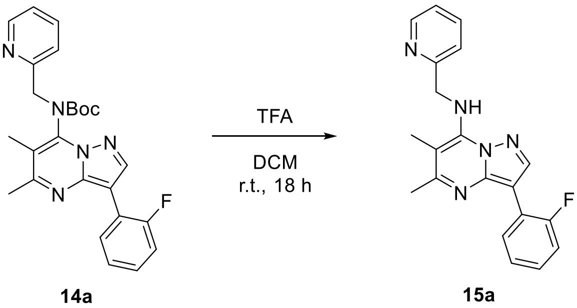

14a **15a**

14a was treated according to **General Procedure 5A**, the crude was purified by column chromatography using 50% ethyl acetate in hexane with 0.1% triethylamine to give **15a** as a white solid (67%): Melting point: 145-147°C; ^{1}H NMR (400 MHz, DMSO) δ 8.64 (td, *J* = 7.7, 1.2 Hz, 1H), 8.56 (d, *J* = 4.5 Hz, 1H), 8.38 (d, *J* = 3.7 Hz, 1H), 7.98 (t, *J* = 6.0 Hz, 1H), 7.79 (td, *J* = 7.7, 1.6 Hz, 1H), 7.39 (d, *J* = 7.9 Hz, 1H), 7.33 – 7.18 (m, 4H), 5.21 (d, *J* = 6.0 Hz, 2H), 2.49 (s, 3H), 2.31 (s, 3H); HRMS (ES) $C_{20}H_{19}N_5F$ $[M+H]^+$ requires 348.1619, found 348.1617; Anal. $C_{20}H_{18}N_5F$ requires C 69.15%, H 5.22%, N 20.16%, found C 69.34%, H 5.16%, N 20.02%.

Synthesis of 3-(3-fluorophenyl)-5,6-dimethyl-N-(pyridin-2-ylmethyl)pyrazolo[1,5-a] pyrimidin-7-amine (15b)

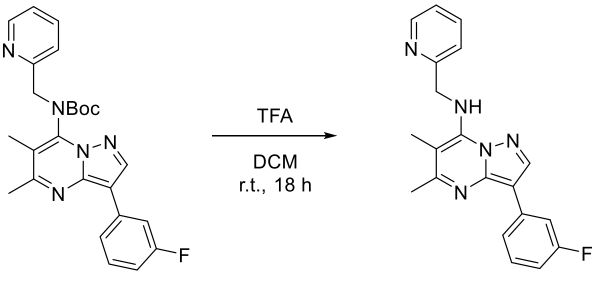

14b was treated according to **General Procedure 5A** to give **15b** as a light brown solid (67%): ^{1}H NMR (400 MHz, DMSO) δ 8.59 (s, 1H), 8.56 (d, *J* = 4.6 Hz, 1H), 8.05 (d, *J* = 11.0 Hz, 1H), 7.98 (d, *J* = 7.8 Hz, 1H), 7.93 (t, *J* = 6.0 Hz, 1H), 7.78 (t, *J* = 7.0 Hz, 1H), 7.44 – 7.35 (m, 2H), 7.32 – 7.26 (m, 1H), 6.94 (td, *J* = 8.6, 2.1 Hz, 1H), 5.21 (d, *J* = 6.0 Hz, 2H), 2.51 (s, 3H), 2.31 (s, 3H); HRMS (ES) $C_{20}H_{19}N_5F$ $[M+H]^+$ requires 348.1619, found 348.1618; Anal. $C_{20}H_{18}N_5F$ requires C 69.15%, H 5.22%, N 20.16%, F 5.47%, found 68.84%, H 5.05%, N 20.05%.

Synthesis of 5,6-dimethyl-N-(pyridin-2-ylmethyl)-3-(4-(trifluoromethoxy)phenyl)pyrazolo [1,5-a]pyrimidin-7-amine (15c)

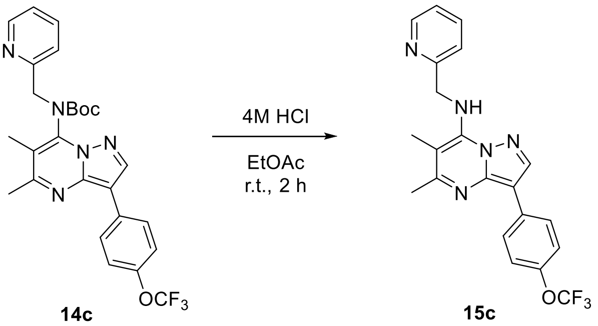

14c was treated according to **General Procedure 5B** to give **15c** as a white solid (82%): ^{1}H NMR (400 MHz, DMSO) δ 8.75 (d, *J* = 5.1 Hz, 1H), 8.44 (s, 1H), 8.28 (t, *J* = 7.3 Hz, 1H), 8.09 (d, *J* = 8.4 Hz, 2H), 7.84 (d, *J* = 7.6 Hz, 1H), 7.72 (t, *J* = 6.2 Hz, 1H), 7.40 (d, *J* = 8.2 Hz, 2H), 5.51 (d, *J* = 2.2 Hz, 2H), 2.55 (s, 3H), 2.29 (s, 3H); LCMS (Method 5-95AB): R_t = 0.797 min / 1.5 min, 100%, MS (ES) $C_{21}H_{19}N_5OF_3$ $[M+H]^+$ requires 414.15, found 414.2.

Synthesis of 5,6-dimethyl-3-(2-(methylsulfonyl)phenyl)-N-(pyridin-2-ylmethyl) pyrazolo [1,5-a]pyrimidin-7-amine (15d)

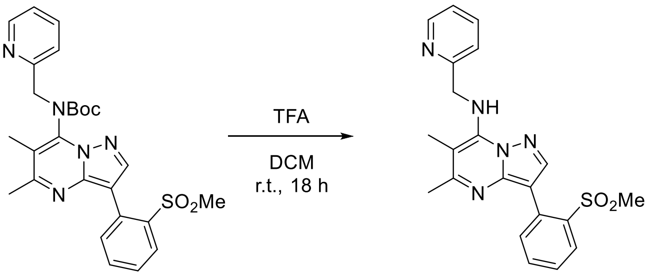

14d **15d**

14d was treated according to **General Procedure 5A**, the crude was triturated with ethyl acetate and diethyl ether to give **15d** as a white solid (49%): Melting point: 116-118°C; ^{1}H NMR (400 MHz, DMSO) δ 8.58 (d, *J* = 4.3 Hz, 1H), 8.23 (s, 1H), 8.10 (d, *J* = 7.9 Hz, 1H), 7.96 (t, *J* = 5.8 Hz, 1H), 7.81 (t, *J* = 7.4 Hz, 1H), 7.75 (t, *J* = 7.5 Hz, 1H), 7.66 (d, *J* = 7.4 Hz, 1H), 7.59 (t, *J* = 7.5 Hz, 1H), 7.43 (d, *J* = 7.8 Hz, 1H), 7.35 – 7.27 (m, 1H), 5.22 (d, *J* = 5.9 Hz, 2H), 2.94 (s, 3H), 2.39 (s, 3H), 2.31 (s, 3H); HRMS (ES) $C_{21}H_{22}N_5O_2S$ $[M+H]^+$ requires 408.1494, found 408.1490; Anal. $C_{21}H_{21}N_5O_2S$ requires C 61.90%, H 5.19%, N 17.19%, found C 61.94%, H 5.43%, N 16.99%.

Synthesis of 5,6-dimethyl-3-(3-(methylsulfonyl)phenyl)-N-(pyridin-2-ylmethyl)pyrazolo [1,5-a]pyrimidin-7-amine (15e)

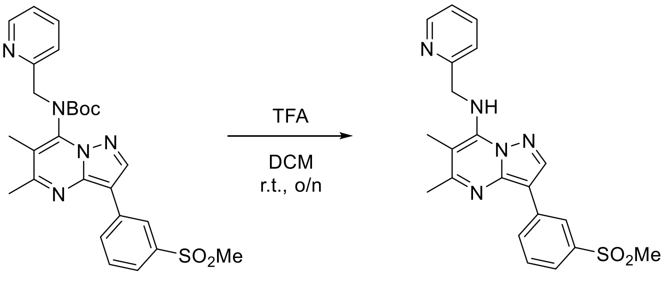

14e **15e**

14e was treated according to **General Procedure 5A**, the crude was triturated with diethyl ether to give **15e** as a white solid (56%): ^{1}H NMR (400 MHz, DMSO) δ 8.73 (s, 1H), 8.70 (s, 1H), 8.57 (d, *J* = 4.0 Hz, 1H), 8.53 (d, *J* = 6.9 Hz, 1H), 8.00 (t, *J* = 5.9 Hz, 1H), 7.79 (td, *J* = 7.7, 1.6 Hz, 1H), 7.71 – 7.62 (m, 2H), 7.39 (d, *J* = 7.9 Hz, 1H), 7.30 (dd, *J* = 6.8, 5.3 Hz, 1H), 5.22 (d, *J* = 6.0 Hz, 2H), 3.27 (s, 3H), 2.52 (s, 3H), 2.32 (s, 3H); HRMS (ES) $C_{21}H_{22}N_5O_2S$ $[M+H]^+$ requires 408.1494, found 408.1495; Anal. $C_{21}H_{21}N_5O_2S$ requires C 61.90%, H 5.19%, N 17.19%, found C 61.73%, H 5.18%, N 16.98%.

Synthesis of 5,6-dimethyl-3-(4-(methylsulfonyl)phenyl)-N-(pyridin-2-ylmethyl)pyrazolo [1,5-a]pyrimidin-7-amine (15f)

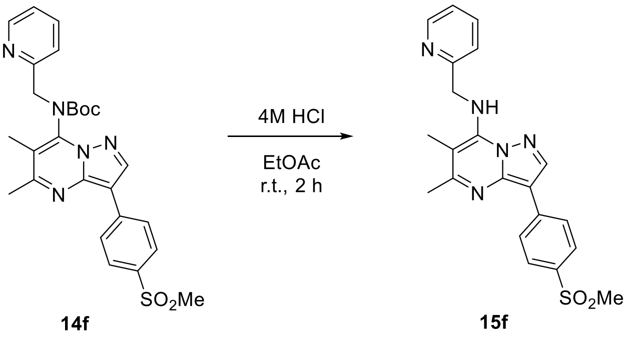

14f was treated according to **General Procedure 5B**, the crude was purified by prep-HPLC to give **15f** as a grey solid (26%): ^{1}H NMR (400 MHz, DMSO) δ 8.69 (s, 1H), 8.55 (d, *J* = 4.1 Hz, 1H), 8.42 (d, *J* = 8.6 Hz, 2H), 7.99 (t, *J* = 6.1 Hz, 1H), 7.89 (d, *J* = 8.6 Hz, 2H), 7.78 (td, *J* = 7.7, 1.8 Hz, 1H), 7.39 (d, *J* = 7.9 Hz, 1H), 7.33 – 7.25 (m, 1H), 5.23 (d, *J* = 6.1 Hz, 2H), 3.20 (s, 3H), 2.53 (s, 3H), 2.32 (s, 3H); LCMS (Method 30-90CD): R_t = 1.055 min / 2 min, 99%, MS (ES) $C_{21}H_{22}N_5O_2S$ $[M+H]^+$ requires 408.15, found 408.1.

Synthesis of 5,6-dimethyl-3-(1-methyl-1H-pyrazol-4-yl)-N-(pyridin-2-ylmethyl)pyrazolo[1,5-a]pyrimidin-7-amine (15g)

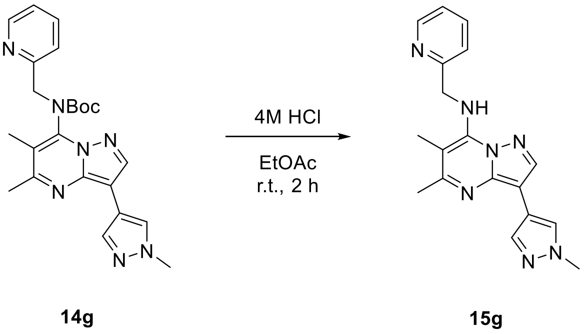

14g was treated according to **General Procedure 5B**, the crude was purified by prep-HPLC to give **15g** as a white solid (37%): ^{1}H NMR (400 MHz, DMSO) δ 8.55 (d, *J* = 4.7 Hz, 1H), 8.23 (s, 1H), 8.08 (s, 1H), 7.88 (s, 1H), 7.82 – 7.73 (m, 2H), 7.36 (d, *J* = 8.1 Hz, 1H), 7.31 – 7.24 (m, 1H), 5.17 (d, *J* = 6.2 Hz, 2H), 3.87 (s, 3H), 2.47 (s, 3H), 2.28 (s, 3H); LCMS (Method 5-95AB): R_t = 0.661 min / 2 min, 99%, MS (ES) $C_{18}H_{20}N_7$ $[M+H]^+$ requires 334.18, found 334.1.

Synthesis of 5,6-dimethyl-3-(pyridin-2-yl)-N-(pyridin-2-ylmethyl)pyrazolo[1,5-a]pyrimidin-7-amine (15h)

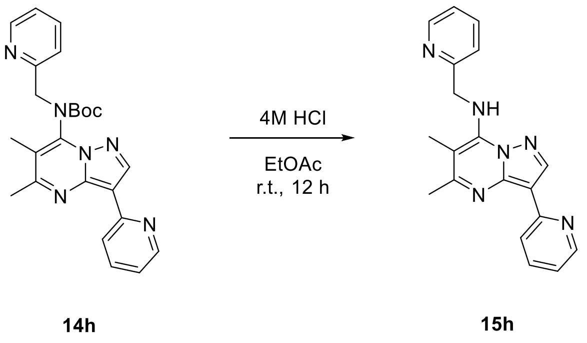

14h was treated according to **General Procedure 5B** to give **15h** as a yellow solid (87%): ^{1}H NMR (400 MHz, DMSO) δ 9.11 (s, 1H), 8.78 (d, *J* = 8.0 Hz, 1H), 8.68 (d, *J* = 5.0 Hz, 1H), 8.64 (d, *J* = 5.8 Hz, 1H), 8.50 – 8.37 (m, 2H), 8.17 – 8.07 (m, 1H), 7.71 (d, *J* = 7.9 Hz, 1H), 7.62 – 7.57 (m, 2H), 5.47 (d, *J* = 5.2 Hz, 2H), 2.61 (s, 3H), 2.33 (s, 3H); LCMS (Method 5-95AB): R_t = 0.680 min / 1.5 min, 99%, MS (ES) $C_{19}H_{19}N_6$ $[M+H]^+$ requires 331.17, found 331.0.

Synthesis of 6-chloro-3-(4-fluorophenyl)-5-methyl-N-(pyridin-2-ylmethyl)pyrazolo[1,5-a]pyrimidin-7-amine (15i)

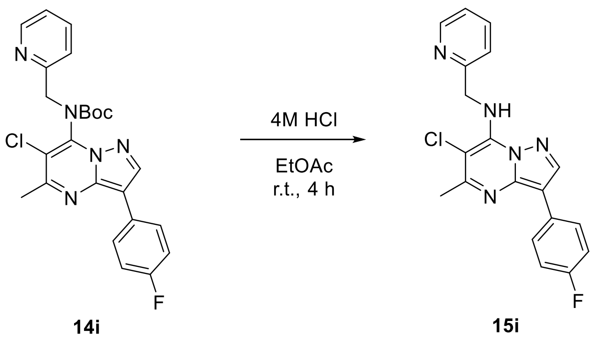

14i was treated according to **General Procedure 5B** to give **15i** as a grey solid (73%): ^{1}H NMR (400 MHz, DMSO) δ 8.59 (s, 1H), 8.53 (d, *J* = 4.3 Hz, 1H), 8.41 (t, *J* = 6.1 Hz, 1H), 8.15 (dd, *J* = 8.8, 5.6 Hz, 2H), 7.79 (td, *J* = 7.7, 1.7 Hz, 1H), 7.38 (d, *J* = 7.9 Hz, 1H), 7.29 (dd, *J* = 6.9, 5.3 Hz, 1H), 7.23 (t, *J* = 8.9 Hz, 2H), 5.38 (d, *J* = 6.1 Hz, 2H), 2.55 (s, 3H); LCMS (Method 5-95AB): R_t = 0.725 min / 1.5 min, 98%, MS (ES) $C_{19}H_{16}N_5FCl$ $[M+H]^+$ requires 368.11, found 368.0.

Synthesis of 5,6-dimethyl-N-(pyridin-2-ylmethyl)-3-(tetrahydro-2H-pyran-4-yl)pyrazolo [1,5-a]pyrimidin-7-amine (15j)

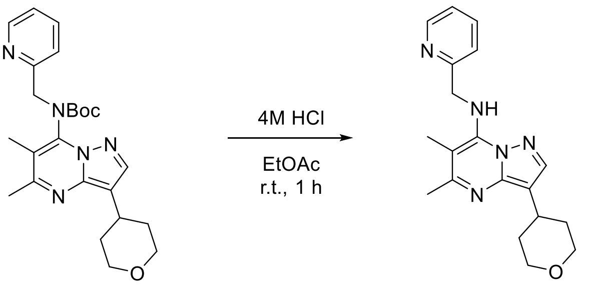

14j **15j**

14j was treated according to **General Procedure 5B**, the crude was purified by prep-TLC using 65% ethyl acetate in petroleum ether to give **15j** as a light yellow solid (57%): ^{1}H NMR (400 MHz, MeOD) δ 8.78 (dd, *J* = 5.7, 0.8 Hz, 1H), 8.48 (t, *J* = 7.2 Hz, 1H), 8.07 (d, *J* = 8.1 Hz, 1H), 7.98 (s, 1H), 7.93 – 7.83 (m, 1H), 5.82 (s, 2H), 4.08 – 3.95 (m, 2H), 3.58 (td, *J* = 11.4, 3.6 Hz, 2H), 3.24 – 3.11 (m, 1H), 2.70 (s, 3H), 2.37 (s, 3H), 1.87 – 1.69 (m, 4H); LCMS (Method 5-95AB): R_t = 0.560 min / 1.5 min, 99%, MS (ES) $C_{19}H_{24}N_5O$ $[M+H]^+$ requires 338.20, found 338.0.

Synthesis of 3-(4-fluorophenyl)-6-methyl-N-(pyridin-2-ylmethyl)pyrazolo[1,5-a]pyrimidin-7-amine (15k)

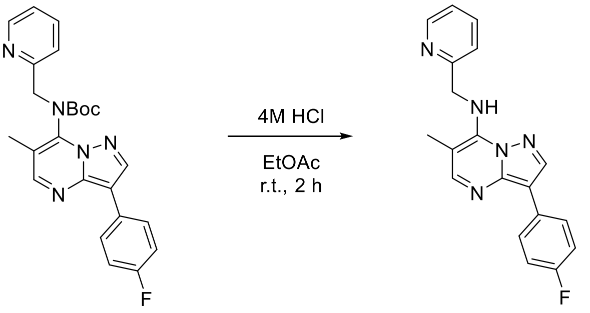

14k **15k**

14k was treated according to **General Procedure 5B**, the crude was triturated with ethyl acetate to give **15k** as a light yellow solid (26%): ^{1}H NMR (400 MHz, DMSO) δ 8.61 (s, 1H), 8.57 (d, *J* = 4.2 Hz, 1H), 8.27 (t, *J* = 6.2 Hz, 1H), 8.19 – 8.14 (m, 2H), 8.12 (s, 1H), 7.80 (td, *J* = 7.7, 1.7 Hz, 1H), 7.38 (d, *J* = 7.9 Hz, 1H), 7.31 (dd, *J* = 6.9, 5.3 Hz, 1H), 7.23 (t, *J* = 9.0 Hz, 2H), 5.18 (d, *J* = 6.3 Hz, 2H), 2.38 (s, 3H); LCMS (Method 0-60AB): R_t = 0.869 min / 1.5 min, 100%, MS (ES) $C_{19}H_{17}N_5F$ $[M+H]^+$ requires 334.15, found 334.2.

Biological testing methods and procedures [1]

Cell Culture

The C6/36 (wAlbB) cell line is a mosquito (*Aedes albopictus*) derived cell line stably infected with *Wolbachia pipientis* (*w*AlbB). To create this cell line, the supernatant from cultured Aa23 cells (*A. albopictus*) naturally infected with the *W. pipientis* strain *w*AlbB was harvested and filtered to remove whole cells. This supernatant was used to inoculate C6/36 cells (ECACC No. 89051705), resulting in a stably *Wolbachia*-infected cell line C6/36 (wAlbB). Cells were incubated at 26 ℃ and subpassaged every 7 days using a 1-in-4 dilution in Leibovitz media (Life Technologies, Loughborough, UK) supplemented with 20% fetal bovine serum (FBS; Fisher Scientific, Loughborough, UK), 2% tryptose phosphate broth (Sigma-Aldrich, Poole, UK), and 1% non-essential amino acids (Sigma-Aldrich).

Anti-*Wolbachia* HCS Assay Setup

C6/36 (*w*AlbB) cells were subpassaged 6–8 days before plating out at a density of 2000 viable cells per well in a 384-well CellCarrier plate (PerkinElmer, Llantrisant, UK), suspended in Leibovitz media with the additives described in the “Cell Culture” section. All compounds were dissolved in DMSO with each compound added to a single well at a final concentration of 5 µM (resulting in <1% final DMSO concentration). Control samples per plate consisted of 12 wells of vehicle control (DMSO) and 6 wells of the following controls: 5 µM doxycycline (positive control, the gold standard for *Wolbachia* reduction; SigmaAldrich) and a suboptimal 50 nM doxycycline concentration. Each well held a final volume of 100 µl, with the exception of the outer wells, which contained 130 µl of phosphate-buffered saline (PBS; SigmaAldrich). After 7 days of 26 ℃ sterile incubation, 25 µl of staining media containing 60 µM of SYTO 11 DNA dye (Life Technologies) was added to each well. After a 15-min incubation, all media was removed from each well and replaced with fresh media (no stain). Using the Operetta high-content automated imaging system (PerkinElmer), five fields per well were imaged using a confocal 60× objective with the Fluorescein filter (excitation filter: 460–490; emission filter: 500–550). The PerkinElmer software Harmony was trained to first identify the cell nucleus and cytoplasm, followed by the spot edge ridge (SER) texture analysis, which was used to score each intact cell on the complexity of the cytoplasm. [1]

Anti-*Wolbachia* HCS Assay Results Analysis

Using the vehicle and positive (*Wolbachia* reduction) controls, a threshold was set to indicate if each cell was classed as infected or uninfected. *Wolbachia*-infected cells (vehicle control)

have a complex cytoplasm texture (high SER texture score), whereas *Wolbachia*-uninfected cells (doxycycline-treated positive control) have a uniform cytoplasm texture (low SER texture score). From this analysis, the following readouts were calculated per well: cell number, SER texture score, and percentage of *Wolbachia*-infected cells. Z' factor (Z') validation of each plate was calculated using the percentage of *Wolbachia*-infected cells value from the vehicle and positive controls. 14 Vehicle controls have a high *Wolbachia* load and therefore a high percentage of cells classed as infected with *Wolbachia*. Positive control (doxycycline-treated) cells have a low *Wolbachia* load and therefore a low percentage of *Wolbachia*-infected cells. All compound sample wells were then analysed and normalized (along with the positive controls) against the vehicle (untreated) control to give a percentage reduction of *Wolbachia*-infected cells. In addition, using the cell number analysis, any compounds with a host cell number amounting to less than 50% of the vehicle control were classed as toxic and retested at a reduced compound concentration. All compounds that were >90% of the positive control's percentage reduction of *Wolbachia*-infected cells were classed as strong hits (because they were similar to or greater than the 5 μM doxycycline positive control). Compounds that yield infection rates between 50% and 90% of the positive control were classed as moderate hits [because they were similar to the suboptimal (50 nM) doxycycline control]. All hit compounds were then reconfirmed in a full dose response to define their potency.

In vitro microfilariae (mf) B. malayi assays [2]

Within the *in vitro* mf assay; compounds are incubated with 8000 mf *B. malayi* per well (five wells per compound) for 6 days, before DNA is extracted and qPCR performed to compare wsp:gst ratio of drug treated vs control wells. Details for the sources of mf B. malayi are described below.

Animals

Male BALB/c SCID were purchased from Harlan Laboratories, UK, while male CB.17 SCID mice and BALB/c WT mice were purchased from Charles River, UK. Male Meriones unguiculatus (Mongolian gerbils; jirds) were purchased from either Charles River, UK or Janvier Laboratories, France. Rodents shipped to REFOTDE, Buea, Cameroon, were maintained in conventional housing with (Halliday *et al.* Parasites & Vectors 2014, 7:472 Page 2 of 14 http://www.parasitesandvectors.com/content/7/1/472) daily cage cleaning and changing of food. Food, water and bedding were sterilised by autoclaving. For *B. malayi* experiments, animals were kept at the Biomedical Services Unit (BSU), University of

Liverpool, UK in specific pathogen-free (SPF) conditions. All experiments carried out in Cameroon were approved by the Animal Care Committee, REFOTDE. All experiments on animals in the UK were approved by the ethical committees of the University of Liverpool and LSTM, and were conducted according to Home Office (UK) requirements. The life cycle of B. malayi (Bm) was maintained in mosquitoes and susceptible Meriones gerbils at LSTM. To generate infective Bm larvae (BmL3) female adult *Aedes aegypti* mosquitoes were fed with Bm microfilariae (mf) collected from infected gerbils by catheterisation, as previously described,[3] followed by mixing with human blood and feeding through an artificial membrane feeder (Hemotek®). Blood-fed mosquitoes were reared for 14 days to allow for development to L3. The L3 were collected from infected mosquitoes by crushing and concentration using a Baermann's apparatus and RPMI medium.

***In vitro* drug metabolism/pharmacokinetic assays**

The DMPK data described in the manuscript were measured once through a high-throughput platform provided by AstraZeneca UK. The methods of the five assays, including LogD7.4, aqueous solubility, plasma protein binding, human microsome and rat hepatocyte clearance measurements, have been reported in detail previously.[4]

Reference:

(1) Clare, R. H.; Cook, D. A. N.; Johnston, K. L.; Ford, L.; Ward, S. A.; Taylor, M. J. Development and Validation of a High-Throughput Anti-Wolbachia Whole-Cell Screen: A Route to Macrofilaricidal Drugs against Onchocerciasis and Lymphatic Filariasis. *J. Biomol. Screen.* **2015**, *20* (1), 64–69.

(2) Halliday, A.; Guimaraes, A. F.; Tyrer, H. E.; Forsbrook, G.; Steven, A.; Cook, D.; Taylor, M. J.; Turner, J. D.; Metuge, H. M.; Patrick, C. N. W.; Arnaud, K.-O. J.; Kwenti, T. D. B.; Enyong, P.; Wanji, S.; Patrick, C. N. W.; Arnaud, K.-O. J.; Kwenti, T. D. B.; Wanji, S. A Murine Macrofilaricide Pre-Clinical Screening Model for Onchocerciasis and Lymphatic Filariasis. *Parasit Vectors* **2014**, *7*, 472.

(3) Griffiths, K. G.; Alworth, L. C.; Harvey, S. B.; Michalski, M. L. Using an Intravenous Catheter to Carry out Abdominal Lavage in the Gerbil. *Lab Anim. (NY).* **2010**, *39* (5), 143–148.

(4) Wernevik J.; Bergström F.; Novén A.; Hulthe J.; Fredlund L.; Addison D.; Holmgren J.; Strömstedt P.-E.; Rehnström E.; Lundbäck T. A Fully Integrated Assay Panel for Early Drug Metabolism and Pharmacokinetics Profiling. *ASSAY and Drug Development Technologies* **2020**, *18* (4), 157-179.
